# Volitional activation of remote place representations with a hippocampal brain-machine interface

**DOI:** 10.1126/science.adh5206

**Published:** 2023-11-02

**Authors:** Chongxi Lai, Shinsuke Tanaka, Timothy D. Harris, Albert K. Lee

**Affiliations:** 1 Janelia Research Campus, Howard Hughes Medical Institute, Ashburn, Virginia, USA.; 2 Howard Hughes Medical Institute and Department of Neurology, Beth Israel Deaconess Medical Center, Boston, MA, USA.

## Abstract

The hippocampus is critical for recollecting and imagining experiences. This is believed to involve voluntarily drawing from hippocampal memory representations of people, events, and places, including the hippocampus’ map-like representations of familiar environments. However, whether representations in such “cognitive maps” can be volitionally accessed is unknown. We developed a brain-machine interface to test if rats can do so by controlling their hippocampal activity in a flexible, goal-directed, and model-based manner. We found that rats can efficiently navigate or direct objects to arbitrary goal locations within a virtual reality arena solely by activating and sustaining appropriate hippocampal representations of remote places. This provides insight into the mechanisms underlying episodic memory recall, mental simulation/planning, and imagination, and opens up possibilities for high-level neural prosthetics utilizing hippocampal representations.

## One Sentence Summary

Rats can volitionally activate non-local spatial representations to perform goal-directed brain-machine interface tasks in virtual reality.

The ability to simulate scenarios in one’s mind is a hallmark of intelligence, facilitating the evaluation of past experiences and future plans. For instance, we can imagine walking around our previous workplace, or imagine how our current workplace might function if we rearranged the furniture. Such imagination requires an internal world model that can be flexibly accessed to construct possible scenarios (1–3).

The hippocampus is a brain region that is critical for memory and imagination (1, 4–6). It holds a model of the environment (also called a cognitive map (7, 8)) that could potentially be mentally traversed for the purpose of recall or simulation. In particular, the hippocampus contains spatial map-like representations of previously explored environments. Each environment’s representation consists of place cells—neurons that fire selectively whenever an animal moves through specific locations (called the “place fields” of those cells) in that environment (9, 10). This results in a distinct multi-cell activity pattern at each location in the environment which, during physical navigation, can be used to decode the animal’s current location from the ongoing pattern of neural activity (11). In contrast, a key aspect of imagination is the activation of neural representations that deviate from current sensory input, such as those that are non-local (i.e., represent locations away from one’s current location). Previous work has shown brief and intermittent activation of non-local hippocampal spatial representations suggestive of the planning of specific paths within a cognitive map (12–21). However, it is unknown whether this activity is volitionally controlled, or rather reflects passive memory-related processes that are presumably non-volitional (22, 23).

To test whether an animal can directly control its hippocampal activity according to its model of the world, we employed a brain-machine interface (BMI) approach because, unlike in humans, we cannot simply ask animals to imagine scenarios. Instead, with BMI methods, we could reward animals for generating neural activity resembling the simulation of specific scenarios. More precisely, we could reward them for the volitional activation of specific non-local representations from the cognitive map—a fundamental building block of scenario simulation. BMI research has a rich history of directly testing for volitional control of activity patterns of neuronal ensembles in motor cortex and related areas (24–35). In the hippocampus, it has been shown that the activity level of individual neurons (36, 37) or the population activity related to individual stimuli (38) can be controlled. However, a real-time BMI that allows humans or animals to control their hippocampal population activity in terms of the content of their cognitive map (e.g., location representations) has never been demonstrated.

## A hippocampal map-based BMI

**Fig. 1.**
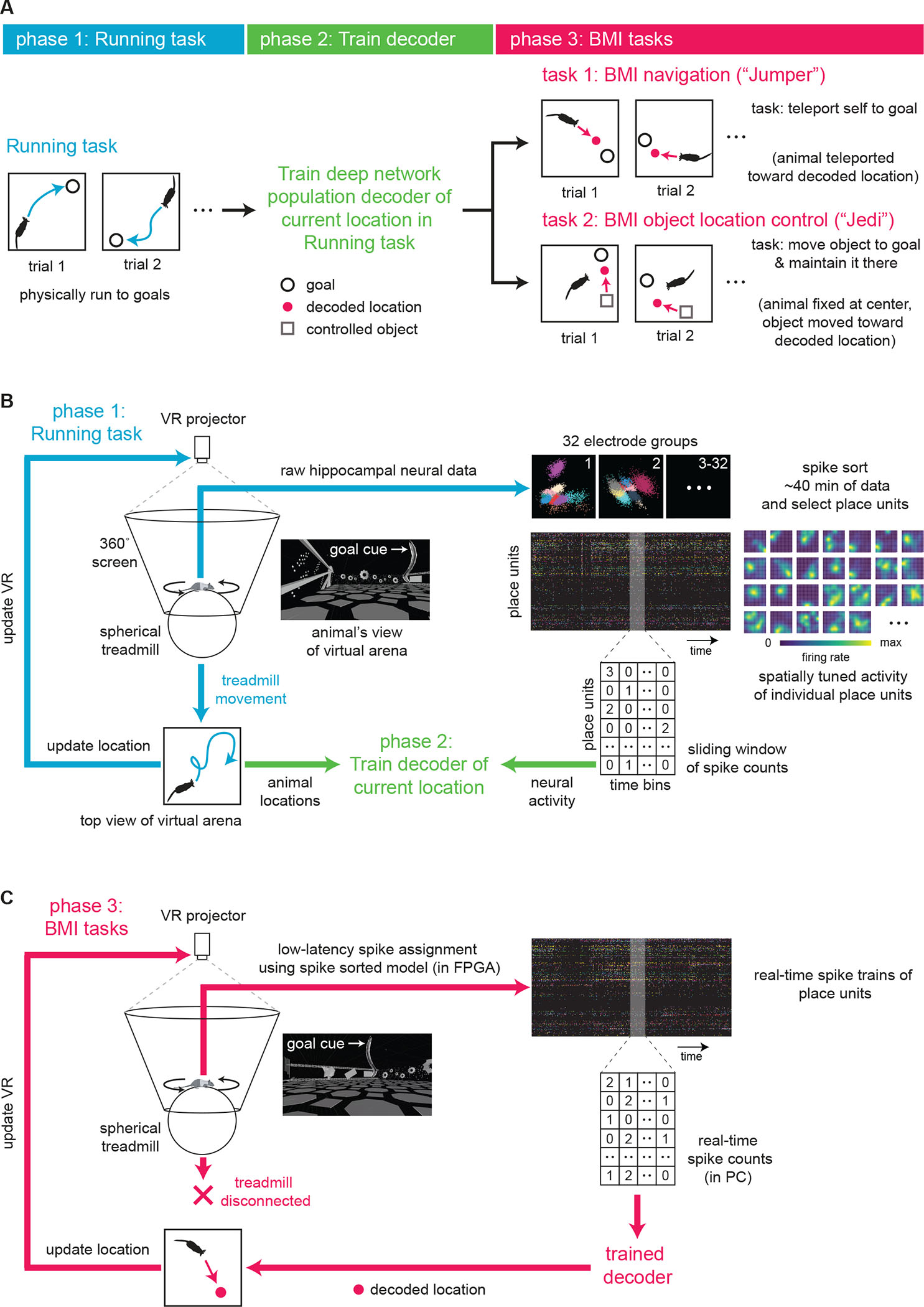
Hippocampal map-based brain-machine interface (BMI) in a virtual reality (VR) system. (**A**) Steps for performing the two different BMI experiments in this study. Rats first physically ran to a series of goals (“Running task”) while their hippocampal neural activity and (virtual) location in a square arena were recorded. This data was used to train a decoder to take neural activity as input and output the animal’s current location in the Running task. In BMI task 1 (“Jumper”), animals needed to generate neural activity that would be decoded as locations they wanted to move to so that they could reach each goal (to obtain reward). In BMI task 2 (“Jedi”), animals were fixed at the center of the virtual arena (but could rotate) and needed to generate activity corresponding to locations where they wanted an external object to move to so that the object reached the goal, then they needed to sustain that activity to maintain the object there (to maximize reward). (**B**) Schematic of VR system (left). Animal was free to rotate its body in the horizontal plane. In the Running task, animal’s location in the virtual arena environment was updated based on treadmill movement. Simultaneously recorded spiking from a population of hippocampal CA1 units expressed place fields—the basis of the cognitive map of the environment (right). Decoder was then trained using binned spiking activity and location data. (**C**) In both BMI tasks, treadmill no longer updated VR. Instead, the animal or object location was controlled solely by real-time hippocampal activity. A neural signal processor rapidly assigned activity to individual units, whose spike counts were fed into the decoder. VR projection was updated based on locations output by the decoder. In the “Jumper” (“Jedi”) task, the animal’s (object’s) virtual location was moved toward the most recent decoded locations.

We designed a real-time hippocampal BMI and two BMI tasks to investigate whether rats could navigate to goals (“Jumper” navigation task), or move external objects to goals while remaining stationary (“Jedi” object location control task), within an immersive virtual reality (VR) environment solely by controlling the activity of a population of its place cells. Each Jumper or Jedi BMI experiment consisted of three phases (Fig. 1A). In phase 1, rats ran to a succession of arbitrary locations marked by a tall, visible goal cue placed in a familiar 2-dimensional virtual arena (“Running task”). Upon reaching each cue, liquid reward was delivered, the trial ended, and the cue moved to another location for the next trial. Animals were secured in a harness and could freely rotate their body and head direction on top of a spherical treadmill (39) while hippocampal CA1 neural activity was recorded (Fig. 1B, fig. S1, movie S1). We applied a recently developed field-programmable gate array (FPGA)-based neural signal processor to perform low-latency (1 ms) assignment of extracellular spikes (recorded from 128 channels) to a population of hippocampal units (40, 41). In the Running task, treadmill movement updated the animal’s location in the virtual environment and many hippocampal units (i.e., place units) displayed spatially modulated activity (39, 42–44) (Fig. 1B, blue arrows) similar to that in real-world environments (8–11). In phase 2, the binned spike counts from the most recent 1.5 or 5 s of activity of these place units and the animal trajectory from the Running task were used to train a decoder (Fig. 1B, green arrows) that estimates the animal’s current location from the neural data every 100 ms. We used a deep neural network for decoding (fig. S2), allowing the use of data augmentation for training—a method that improves the decoder’s performance given limited data as well as its noise robustness. In phase 3, the treadmill was disconnected from the VR system and the animal’s ability to control its own or an object’s translational movement was limited to controlling its hippocampal activity, which was converted by the decoder into a specific location output every 100 ms (Fig. 1C). Importantly, the decoder was trained to estimate the animal’s current location in the Running task only, not its location in the subsequent BMI tasks. During BMI periods, the animal needed to generate activity corresponding to locations away from its current location.

## BMI navigation task

**Fig. 2.**
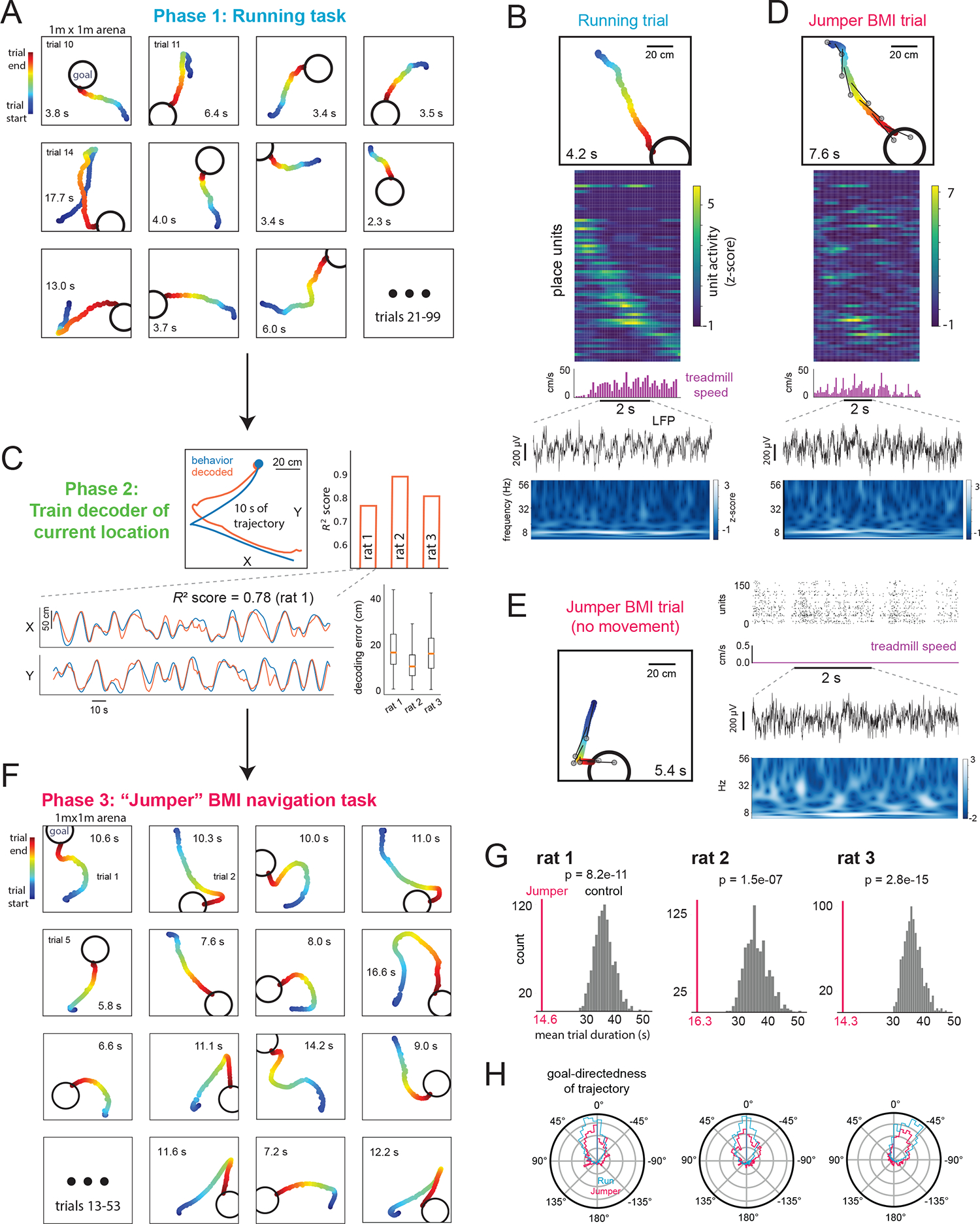
Rats can navigate to goals by controlling their hippocampal activity. In both Running and Jumper BMI tasks, animals were rewarded when they reached each goal. (**A**) Animal trajectories in virtual arena for consecutive Running task trials. Trial duration (time to reach goal) in seconds shown. (**B**) Example Running task trial. From top: trajectory, firing rate (z-scored) of individual units (units were ordered by time of peak activity), treadmill speed, and LFP from one recording channel and corresponding wavelet spectrogram during trial. (**C**) Accuracy of trained decoder of animal’s current location for held-out Running task data. Actual and decoded trajectories during example trial (top left) and across several trials (for X and Y coordinates separately, bottom left). Median decoding error (distance between actual and decoded locations) with range and quartiles (bottom right). (**D**) Example Jumper BMI trial with similar trajectory as Running trial in (B). From top: trajectory generated by the animal controlling its hippocampal activity and the decoder output (animal is teleported toward decoded location; each gray circle represents the decoded location at the time the animal is at the corresponding point in the trajectory connected by the dark line, sampled here every 1 s), firing rate of individual units (using same order of units as in (B)), treadmill speed, LFP, and spectrogram. (**E**) Example Jumper BMI trial in which animal did not move the treadmill. Trajectory as in (D) (left). Right, from top: unit activity, treadmill speed, LFP, and spectrogram. See fig. S10 for all 10 non-movement trials. (**F**) BMI-generated trajectories for consecutive Jumper trials. (**G**) Mean Jumper trial duration (vertical line) is significantly lower than distribution of expected mean duration for simulated trials if goals were in random locations. (**H**) Polar distribution of angle between direction of movement and direction to goal during Running and Jumper tasks. Zero corresponds to animal movement directly toward the goal center.

In the Jumper task, we tested whether animals could navigate to arbitrary goal locations as in the Running task, except here via BMI-based first-person teleportation. After rats performed the Running task for ~40 min (~120 trials) (Fig. 2A,B, movie S1), the data were used to train the decoder, which accurately estimated the rat’s current location in the Running task (validation set R^2^=0.78–0.88, Fig. 2C). Jumper trials were identical to Running trials, except the animal’s location was updated to the BMI-decoded location (smoothed with a 3 s sliding window to help reduce potential high-frequency visual jitter of the VR updates) (Fig. 2D,E, movie S1). If an animal did not reach the goal within 62 seconds, the trial ended and a new goal cue appeared at a random location.

Rats successfully navigated by controlling their hippocampus, generating efficient paths to each goal (Fig. 2F, see fig. S3–S5 for all trials of 3 rats, and fig. S6–S8 for all trials re-decoded using a shorter decoding window and without smoothing). To check if this performance could be due to non-spatially-specific neural activity (e.g., modulating global firing rate), we randomly shuffled the spike trains across place units, ran the shuffled data through the original decoder to produce simulated trajectories, then determined how long it would have taken to reach the same sequence of goal locations as in the original experiment. Shuffled-unit mean trial durations were much longer than the actual means (*P*<10^−100^, 3 rats, 1 session each), suggesting performance depended on generating place field-related activity. To test whether generating non-goal-directed sequences of location-specific activity (e.g., random movement within the cognitive map) could explain the performance, we randomly shuffled the goal locations in each trial while preserving the original BMI trajectories, then determined the time that would have been needed to reach the shuffled goals. Shuffled-goal mean trial durations were again much longer than actual means (*P*=2.8×10^−15^-1.5×10^−7^, Fig. 2G), indicating animals’ BMI trajectories were clearly goal-directed. Goal-directedness was also apparent from the distribution of angles between the animal’s instantaneous direction of BMI-generated movement and the direction from the animal’s current location to the goal, which was concentrated around a value near 0° (Fig. 2H). Thus, even though Jumper trials took longer than Running trials (mean trial duration across animals: 15.1 versus 6.9 s; note, though, that BMI decoding and smoothing added a few seconds to Jumper durations), the animals’ routes revealed effective, goal-directed, map-based BMI navigation. Furthermore, such performance was achieved without extensive BMI training (Fig. 2F, fig. S3–S5 show sessions 3, 9, 2 for rats 1–3, respectively, Table S1; a fourth rat failed to perform either BMI task).

While animals were free to physically run during the Jumper task, such movement was not necessary for task performance. Initially, animals ran as in the Running task, but in later trials animals ran less (fig. S9). In a subset of trials (10 out of 161 trials) (Fig. 2E and fig. S10), animals remained still, yet in all cases they efficiently reached the goal. Moreover, this successful navigation did not depend on activity in population burst events (PBEs), which often appear during immobility, and during which brief activation of place cell representations for remote locations has been shown to occur (13–16, 18, 20, 21, 23).

## BMI object location control task

While episodic memories are encoded and often retrieved using a first-person perspective, individuals can also imagine scenarios from a third-person perspective, with other animate and inanimate players taking part. Furthermore, imagination often involves holding a single thought in mind for extended periods. Therefore, our second BMI task, Jedi, tested whether animals—while remaining in the same place—could use the same map of the arena to control the location of a virtual object, guide it to the goal cue location, and maintain it nearby. Jumper and Jedi thus employed different forms of feedback: self-location and the location of an object, respectively. After the same Running task and decoder training phases as in Jumper, the animals in Jedi were fixed (but could freely turn) at the arena’s center and the object’s location was updated to the BMI-decoded location (with a 2 s smoothing window). In each trial, the goal cue remained in the same place, providing reward as long as the object touched it. After 3 min or the rat received 0.5 mL reward, whichever came first, a new goal cue appeared at a distant random location for the next trial.

**Fig. 3.**
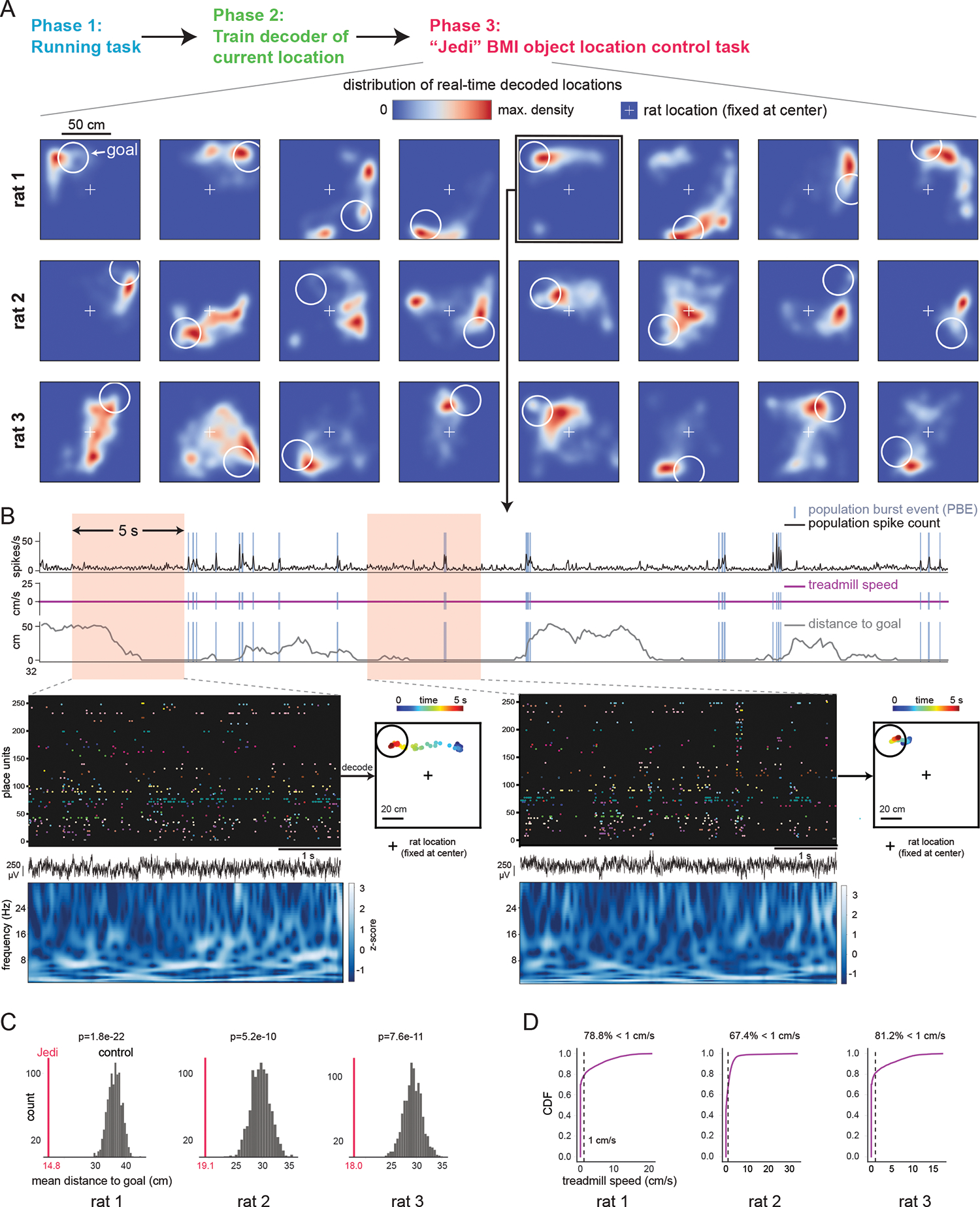
Rats can move objects to remote goal locations and maintain them there by controlling their hippocampal activity. In the Jedi BMI task, trials did not end when the external, controlled object first reached the goal; instead, animals were rewarded as long as the object was in the goal region (white circle), for up to 3 min per trial. The animal was always fixed at center of virtual arena, but could rotate its body and generally turned toward each goal. (**A**) Distribution of real-time decoded locations (output every 100 ms) generated by the animal controlling its hippocampal activity across 8 consecutive Jedi BMI trials for rats 1–3. Panels show decoded locations during each trial (up to 3 min, fig. S11). Periods when animal’s body rotated >12°/s were excluded. See text and methods for details. The external, controlled object (which was visible for rats 1–2, invisible for rat 3) was moved toward the decoded location (fig. S11 shows that the distribution of object locations was essentially the same as the distribution of decoded locations). (**B**) A 40-second-long period during an example trial during which animal did not move the treadmill. From top: Summed activity across all units with population burst events (PBEs) identified, treadmill speed, distance of decoded location from goal (0 means inside goal region), and close-ups of two 5-second periods (left: as animal moves object to goal; right: as animal maintains object at goal; points in arena represent sequence of decoded locations) with spike trains of units, LFP, and spectrogram. See fig. S12 for additional example periods. (**C**) Mean distance of decoded location from goal across all trials (vertical line) is significantly lower than mean distance expected for randomized goal locations. (**D**) Treadmill speed distribution during periods shown in (A) showing animal was generally still during task performance.

Rats could activate and sustain a remote location’s representation around the goal for long periods until the trial ended, then shift attention to the next goal (Fig. 3A,B, fig. S11, movie S1). Performance was measured using the mean distance (over time) between the decoded locations and goals. Shuffling spike trains across units yielded much greater mean distances than the actual means (*P*=2.2×10^−5^-2.6×10^−3^, 3 rats, 1 session each). To assess the goal-directedness of BMI-generated activity, we shuffled the goal locations while preserving the locations output by the decoder. The decoded (and controlled object’s) location was far more concentrated around the actual remote goal cue than shuffled goal locations (*P*=1.8×10^−22^-5.2×10^−10^, Fig. 3C, fig. S11), indicating clear goal-directed control of activity. Again, such performance occurred without extensive training (Fig. 3A shows sessions 7, 6, and 3 for animals 1–3, respectively). Task performance was not dependent on PBEs, as there was no change in performance when all activity in PBEs was eliminated and the decoder was re-run post-hoc (fig. S11).

Animal movement was generally low when engaged in the Jedi task (fig. 3D) and movement was not required for successful performance. There were many longer periods (≥8 s long with treadmill speed ≤1 cm/s, 38 periods, mean 17.3 s, maximum 44.0 s) during which the animal did not move the treadmill while it directed the object to the goal and/or held it there (34 of 38 periods, Fig. 3B, fig. S12). Activity during PBEs was also generally not necessary for performance in these non-movement segments (fig. S12).

## Features of volitionally generated spiking and local field potential activity

What characteristics did the volitionally generated activity have? First, mean firing rates per unit were similar between Jumper and Running tasks (fig. S13A). Mean firing rates per unit were correlated across Jedi and Running tasks, but lower in Jedi (fig. S13B)—consistent with the decreased physical movement in Jedi.

**Fig. 4.**
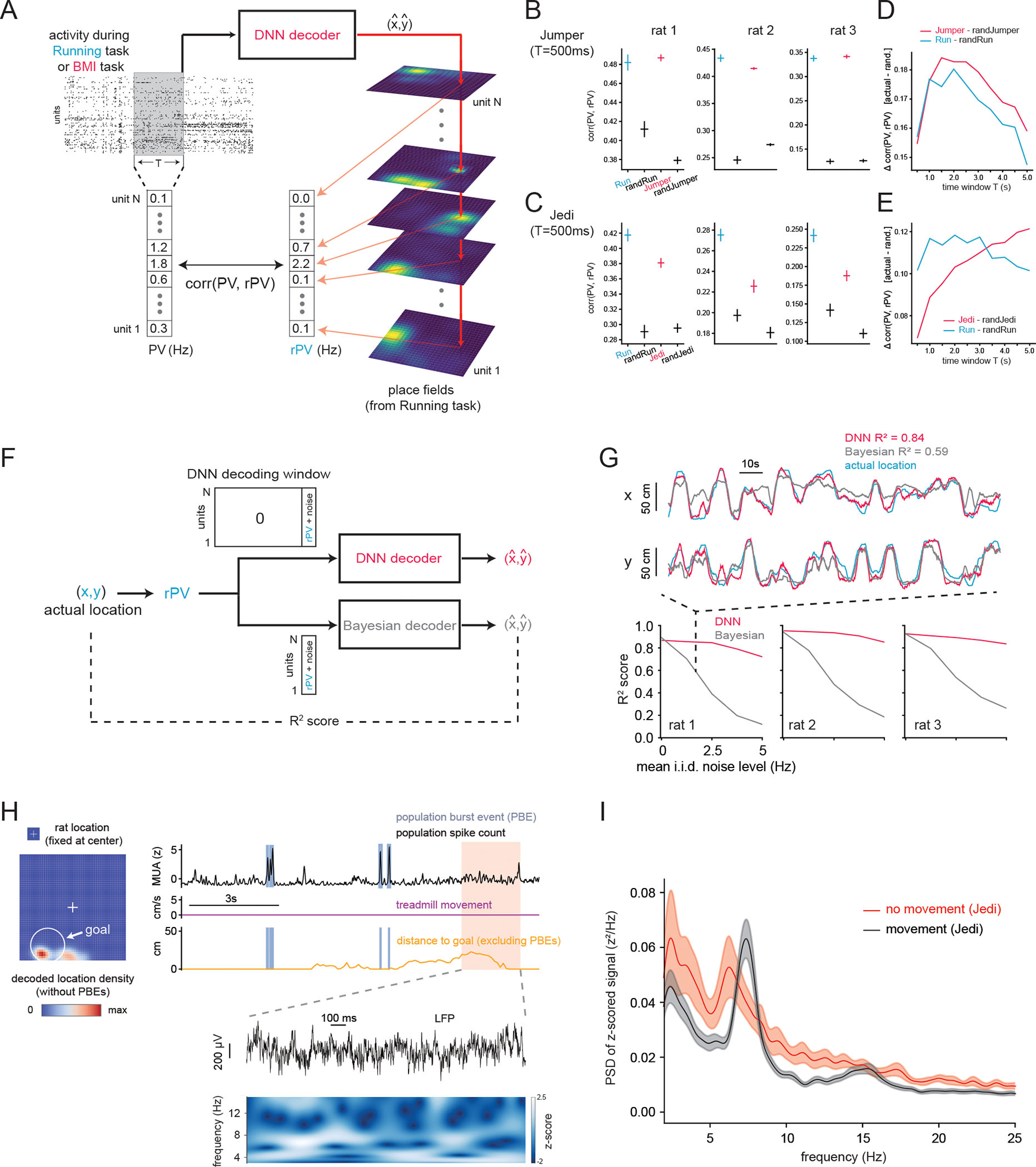
Volitionally generated non-local activity is similar to the activity when the animal is at the corresponding locations and is associated with theta-band power in the LFP. (**A**)-(**E**) The population vector (PV) of ongoing spiking activity was compared to the average place field activity (rPV) at a given location during the Running task. (**A**) Schematic of comparison. (**B**) Mean correlation of instantaneous (500 ms window) PV during Running or Jumper task with rPV for the current location (in Running task), current decoded location (in Jumper task), or random location in Running (randRun) or Jumper (randJumper) task. (**C**) Same as (B) but for Jedi task. For Jedi, only periods when decoded location was near (within 5 cm of) goal were included (also for (E)). (**D**)-(**E**) Correlation of PV with rPV relative to baseline random value as a function of time integration window for determining the PV. (**F**)-(**G**) Evaluation of decoder performance when ground truth activity for each location, i.e., the rPV, was input into decoder. (**F**) Schematic of evaluation procedure. (**G**) Comparison of our DNN decoder to Bayesian decoder for different levels of added noise, with example traces using a specific level of noise (top). (**H**) Distribution of decoded location (left) during Jedi task segment with no treadmill movement (right). Right, from top: Summed activity across all units with population burst events (PBEs) identified, treadmill speed, distance of decoded location (excluding data during PBEs) from goal, and close-up LFP with spectrogram. (**I**) Power spectral density of z-scored (for pooling across animals) LFP during Jedi task for periods of treadmill movement and all long segments (≥8 s) without treadmill movement. See text and methods for details. Here and elsewhere all CIs are 95% CIs.

We then investigated the hypothesis that, to move itself or the object toward a given (decoded) location in the Jumper and Jedi tasks, animals generated a pattern of firing rates across units (i.e., a population vector, or PV) similar to the mean PV at that location over the entire Running task (called the reference PV, or rPV) (Fig. 4A). (Note that the set of rPVs for all locations is thus equivalent to the standard place field map across the population.) We examined the correlation between the PV generated at each moment (in every 500 ms window) during Jumper or Jedi and the rPV of the decoded location at that moment. As a benchmark, we computed the correlation between the 500 ms PVs during the Running task with the rPVs corresponding to the animal’s actual locations at those times (Fig. 4B,C “Run”), as well as the correlation between the Running task PVs and the rPVs of random locations (Fig. 4B,C “randRun”). We then correlated Jumper or Jedi PVs with the rPVs of the decoded locations at each moment (Fig. 4B for “Jumper”, Fig. 4C and fig. S14 for “Jedi”), and with rPVs of random locations (“randJumper”, “randJedi”). Jumper and Jedi PVs were significantly correlated with the rPVs associated with the decoded locations versus random locations, consistent with the hypothesis. Furthermore, Jumper PV-rPV correlations were comparable to Running task PV-rPV correlations. In line with this, the example Running (Fig. 2B) and Jumper (Fig. 2D) trials, which happened to share similar trajectories, showed similar activity patterns across place units over time. PV-rPV correlation scores were, unlike in Jumper, lower in Jedi than the Running task using 500 ms windows (Fig. 4C), consistent with noisier generation of non-local representations and/or lower firing rates (fig. S13B) in Jedi. However, with longer integration windows (>500 ms) (Fig. 4D,E), the PVs generated during Jedi matched the rPVs as well as the best match during the Running task (note that longer integration times work for Jedi because animals activated goal location representations for extended periods). These results indicate that, during BMI task performance, animals generated non-local population activity as similar to the corresponding place field representations as when they actually visited those locations in the Running task. Were these place field (i.e., rPV)-like patterns what our deep network detected to decode location? While determining what features a deep network uses for decoding is generally not straightforward, inputting a single location’s rPV for a brief duration was sufficient to produce accurate location decoding (Fig. 4F–G), consistent with the decoder being tuned to detect rPV-like activity. In addition, unlike the commonly used Bayesian decoder (45), our decoder was highly robust to noise (Fig. 4G) by design due to the use of data augmentation during training.

Lastly, we analyzed the local field potential (LFP) activity during BMI task performance (Fig. 4H,I). When animals move, the rodent hippocampal LFP is known to display prominent theta band (~5–12 Hz) power, which peaked at 7.3 Hz during periods of movement in the Running and BMI tasks (Fig. 4I). During the extended periods of non-movement when the animal was performing the Jedi task, the theta peak shifted down to 6.3 Hz (Fig. 4I). Note that, unlike the more continuous theta oscillations during movement, the oscillations during such non-movement periods tended to be more intermittent.

## Discussion

Previous BMI research has yielded major advances in the control of robotic arms, computer cursors, and other devices by activity from primary motor, premotor, and posterior parietal cortex (24–35). The hippocampal cognitive map has a code that represents space in terms of absolute location in the external environment versus location relative to (e.g., in front of, or to the right or left of) the animal (8–11), and it was unknown whether a subject could control a BMI based on this code. Here, we demonstrated a hippocampal map-based BMI in which the subject is able to control its location or that of other objects by activating location representations in terms of absolute space, independent of where the animal currently is. That is, even though animals generally (but not always) turned their body toward the goal, the activity that needed to be generated differed depending on the location of the goal with respect to the environment. The relatively small amount of training needed for the animals to perform our BMI tasks is in line with our use of a biomimetic decoder (35, 46), i.e., one based on the neural code that the subject naturally employs.

In humans, imagining or recalling objects or video clips is accompanied by hippocampal activity in individual neurons similar to that when viewing the original stimuli (47, 48). This suggests that the mechanisms allowing animals to selectively activate their non-local hippocampal spatial representations as we have shown here could also underlie our ability to actively recall or imagine experiences in other places. The ability of rodents to perform these BMI tasks should thus allow imagination, as well as the voluntary recall of memory, to be investigated using the range of tools available for this model system. More generally, the neural processes engaged here could underlie our capacity to perform “mental time travel”, i.e., travel back in time by re-experiencing richly detailed episodic memories and forward in time by generating possible future scenarios (49). Mental time travel depends critically on the hippocampus (4–6, 50–52) and enables subjects to internally simulate new experiences according to their world model. This can aid decision-making and facilitate learning in complex situations where trial and error is expensive, as shown using artificial agents (*3, 53*–55).

Along these lines, our animals could control their hippocampal map-based activity on a timescale of seconds, corresponding to the speed and duration at which humans relive past events or imagine new scenarios. Navigational trajectories each lasted ~10 s, and a virtual object could be held at a remote location for several seconds. This contrasts with the previously described fast (~100 ms) sequences of non-local hippocampal activity in awake rodents (i.e., awake replay events, which are associated with population bursts and sharp wave-ripples) thought to be associated with planning (12, 16, 18, 21), and which were not responsible for the performance in our BMI tasks (analysis in which all PBEs were removed). The content of such replay events, which can portray specific routes through the environment starting from the animal’s current location, has been shown to be correlated with deliberative (12) and future (16, 18, 21) behavior. However, it is not known whether this content is—or replay content in general can be—under an animal’s volitional control. For instance, hippocampal activity displays similar fast sequences during sleep (23), thus non-local path generation per se does not appear to require intention. If awake replay is volitionally controlled, these events could represent a brief consideration of alternatives for making a quick decision and be distinct from the more comprehensive mental simulations of possible scenarios that take seconds. Previous work has also described neurons in the hippocampus and related areas whose activity is tuned to the angle to a goal or salient cue/object relative to the direction the animal is facing (56–59). In addition, hippocampal neurons that are tuned to the location of conspecifics have been found (60, 61). As with fast sequences, whether these forms of activity that reflect locations away from the animal are volitionally controlled is yet to be determined.

Beyond aiding decision-making, the ability to control the content of the hippocampal spatial and episodic memory system could help explain the richness of our inner lives. Finally, the ability to control hippocampal activity to guide oneself or objects to intended locations—and do so with high signal-to-noise read out using our decoder—could lead to new BMI applications for restoring or enhancing function based on realizing a subject’s high-level intentions with respect to their internal world models.

## Supplementary Material

Movie S1

Supplementary Material including caption for Movie S1

## Data Availability

All data needed to assess the findings in this study are publicly available at Zenodo (62). All analysis methods are described in the main text and supplementary materials, and the code for building and applying the decoder is publicly available at Zenodo (62).
