# Supplementary material for: Volitional activation of remote place representations with a hippocampal brain-machine interface
Source: Science. Author manuscript; Available in PMC 2023 Nov 28. (PMC10683874; doi:10.1126/science.adh5206)
Supplement: Supplementary Material including caption for Movie S1 [file NIHMS1942713-supplement-Supplementary_Material_including_caption_for_Movie_S1.pdf]

Supplementary Materials for

Volitional activation of remote place representations with a hippocampal brain-machine interface

Chongxi Lai*, Shinsuke Tanaka*, Timothy D. Harris, Albert K. Lee

Correspondence to: chongxi.lai@gmail.com, alee31@bidmc.harvard.edu

This file includes:

Materials and Methods

Figs. S1 to S14

Table S1

Caption for Movie S1

Other Supplementary Materials for this manuscript include the following:

Movie S1

Materials and Methods

Subjects

The subjects were adult male Long-Evans rats, weighing ~350-450 g at the time of surgery. Animals were individually housed in home cages fitted with custom-made running wheels throughout training and after surgery on a 12 h light / 12 h dark schedule. Animals were water-restricted to provide motivation to perform the Running and BMI tasks, during which liquid reward could be obtained. All procedures were performed according to the Janelia Research Campus Institutional Animal Care and Use Committee guidelines on animal welfare.

Virtual reality system

Virtual environment software and hardware

Our custom virtual reality (VR) software was developed as part of Janelia's virtual reality software platform (Jovian). Our software suite, named 'MouseoVeR', was written in C++ and built from a number of open-source software components (Boost, Bullet, osgBullet, osgWorks, OpenSceneGraph, Collada, OpenGL, and Qt) (*63*). Virtual arena environments were created using the open-source animation software Blender (www.blender.org) and rendered by MouseoVeR. Blender environments were rendered using six virtual camera objects located at a single point in space to cover all the directions of a cube. For display, the six images were converted to an annulus shape to be projected from above onto a custom-made screen (80% polyester, 20% spandex, First Response Custom Sewing, Inc.). The screen was shaped like an inverted truncated cone stretched between two aluminum rings (top and bottom ring diameters: 122 cm and 63.5 cm, respectively, height: 100 cm). The final image encompassed a viewing angle of 50° above and 30° below the horizontal eye level. The top ring was mounted on an aluminum frame. The top of the screen was covered by a horizontal sheet of transparent acrylic (122 × 122 × 1.2 cm). A projector (InFocus 5312a) with short-throw lens (InFocus LENS-060) was mounted horizontally on the ceiling, and images (1600 × 1200 pixels) of a virtual environment were reflected at a right angle off a round mirror (18-inch diameter, McMaster-Carr) onto the screen through the acrylic sheet. The cone-shaped screen surrounded a spherical treadmill, which consisted of a large, hollow, lightweight polystyrene sphere (24 in diameter, 350 g total when the 2 separate hemispheres were glued together, Foam Mart ball modified by WeCutFoam) resting on a bed of seven individually air-cushioned ping-pong balls (30 psi) arranged around the lower half of the sphere by an acrylic frame (https://www.janelia.org/open-science/large-spherical-treadmill-rodents (*63*)). To prevent chewing of the sphere by the rats, the sphere was covered with packing tape (Scotch). Rotation of the sphere around its vertical axis was prevented by four "yaw blockers" (small, vertically oriented rubber wheels with custom made attachments) separated by 90° around the sphere's equator, thus the animals could only change direction by rotating themselves on the treadmill. To track the motion of the treadmill, two cameras separated by 90° were positioned at the equator and focused on 4 mm^2 regions under infrared illumination (modified from FlyFizz, (*64*)). The cameras captured 30 × 30 images of the treadmill surface at 4 kHz and the motion of the treadmill was computed from the accumulated differences in the images over time. In each iteration of the rendering loop, MouseoVeR communicates with the treadmill's data server to retrieve the updated motion

values since the last request. For calibration, we created mappings between 180° rotations of the treadmill along each of the two relevant directions in real-world space and the data server's coordinate space. Body orientation in the horizontal plane was detected by a rotational encoder (TRD-MX1000AD, Koyo) that was calibrated by a photointerruptor (EE-SX672-WR, Omron) and a Teensy 3.1 microcontroller. The animal was held in place at the apex of the treadmill by a body harness, consisting of a jacket for the forelimbs (Harvard Apparatus), two spandex belts supporting the lower body (16 × 1.5 cm), and a neoprene backbone (10.5 × 5.5 cm, 70A hardness). A 3D-printed hook on this backbone was used to fasten the animal to an aluminum arm containing two hinges, which permitted a small amount of up-and-down body movement during walking. The arm was attached to a bearing (X-contact, 3.500 × 4.000 × 0.25, Swerve Drive Specials) on the bottom acrylic plate of a 128-channel motorized commutator (Saturn, Neuralynx), which allowed the rat to freely turn its body in any horizontal direction on the treadmill (i.e., 360° free rotation with no restriction on the number of net turns it could accumulate in either the clockwise or counterclockwise direction). Another 3D-printed arm that also rotated with the animal was attached to hold a lick port in front of the animal. To not block the animal's forward view, this arm had an open window (16 × 12 cm) in front of the animal. On the top of the commutator, a liquid rotary joint (Doric) was mounted, and its rotation was linked to the commutator so that the water reward supply line could go through the commutator and freely rotate with it. The back end of the water supply line was connected to two solenoid valves (EV-2-12, Clippard) and a syringe pump (PHD2000, Harvard Apparatus) to control the amount and timing of water reward. In addition, a 50 mL reservoir and a small solenoid valve (SV-2C-12-3-V, Clippard) were mounted onto the aluminum arm so that sweetened water (Kool-Aid) could also be provided, if desired. Whenever reward was delivered, a buzzer beeped (SunFounder, 2300 ± 300 Hz). On the acrylic frame holding the spherical treadmill, four nozzles (Eppendorf 1000 μL tips connected to stainless steel tubing) were attached 30 cm away from the animal to supply airpuffs (30 psi). Airpuffs were triggered manually when animals did unfavorable behaviors, such as chewing the equipment or sitting still for too long (e.g., during Running task training). The screen, projection, yaw restriction, commutator, and body harness systems were modeled after (*39*).

VR game engine for task implementation

The Running, Jumper, and Jedi tasks were all written in Python using our VR game engine called Playground. Playground (https://github.com/chongxi/playground) is a Python-based software system that allows for the creation, execution, and control of complex behavioral tasks in VR environments. At the core of Playground is a Finite State Machine (FSM) framework used to define the task's logic and rules. For instance, when a trial starts, the task state is set to "trial started". A cue is then generated at a random location. Once the animal moves close enough to the goal, the cue triggers a transition in the task state from "trial started" to "reward cue touched", then to "reward delivering", and finally to "trial finished". Each state transition can lead to a specific outcome. The state transition and associated outcome is fully defined in the FSM. This FSM is fully customizable and simple to prototype with using a few lines of Python code, allowing for the creation of a wide range of tasks, from simple go/no-go paradigms to more complex, multi-state tasks. Playground also includes a user-friendly visualization module that enables researchers to intuitively track task states, animal behavior inside a 3D-modeled environment, and electrophysiological data, such as spike trains and waveform features,

simultaneously in real time. With this fast 3D visualization, researchers can observe and understand the relationship between behavior, neural activity, and task states for rapid BMI task prototyping. Playground also integrates with the Jovian VR platform, which is used in this study to render the 360° immersive virtual environment. The combination of Playground and Jovian software allows for the precise control and manipulation of the virtual environment, including the ability to teleport objects and change their properties in real time according to either animal behavior or BMI output. The code for all of the tasks that were used in this study can be found on the GitHub page of the Playground project (https://github.com/chongxi/playground/blob/master/playground/base/task/task.py).

Brain-machine interface system

Overview

Our brain-machine interface system performs real-time analysis and converts the place unit activity of a population of CA1 units into an estimate of the animal's current location in the Running task (and the "desired" location in the BMI tasks). It consists of a Field Programmable Gate Array (FPGA)-based neural signal processor (NSP) and a deep neural network (DNN)-based decoder, the latter of which resides on a personal computer (PC). The NSP is connected to up to 5 32-channel headstages (RHD2132, Intan Technologies) that amplify and sample neural signals from up to 160 channels (128 channels were used in this experiment) at a rate of 25 kHz per channel. The NSP communicates with the PC through Python application programming interfaces (APIs), which allow the NSP to retrieve the parameters of a spike sorting model from the PC. The FPGA uses these parameters to classify spikes as belonging to individual units in 1 ms, and sends the assigned spike-id's (which identify the units) along with spike timestamps to the PC through a low-latency interface for real-time decoding. The FPGA-based NSP is described in more detail below in this section, and the DNN-based decoder in the BMI task section.

Our BMI system was integrated with the VR systems. During the pre-BMI Running task, band-pass filtered raw recordings, together with online-detected spike waveforms, the waveform features, and their electrode origins, as well as the animal's location and body orientation at each moment, were collected and stored. After conducting semi-automated spike sorting, place units were selected based on their activity level and spatial information for use in training the decoder. This decoder was trained to estimate the animal's current location in the Running task based on recent neural activity from the population of place units. This decoder was then deployed in real time for BMI tasks. In the BMI tasks, the online-decoded location was transmitted to the VR game engine, enabling updates of the task state, and from the game engine to MouseoVeR for VR rendering.

FPGA-based NSP for on-chip spike assignment

The real-time FPGA-based neural signal processor for classifying spikes into their source unit is described in detail in (*40*). Briefly, the 128 channels of raw data that were amplified and sampled at 25 kHz per channel (32 bits per sample) by the headstages were input into the FPGA (KC705, Xilinx). Inside the FPGA, (1) each channel was band-pass filtered between 500-3000 Hz using a custom pseudo-linear phase FIR filter, (2) common noise across

channels was removed by reference subtraction, (3) the data were split into 32 independent 4-channel electrode groups (2 per 8-site shank), (4) within each electrode group, spikes were detected using an amplitude threshold (see below) and 19 points (0.76 ms) per channel around each spike's peak were assigned as the (4 × 19 dimensional) waveform for that spike, (5) principal components of each spike waveform were extracted as features, based on principal component analysis (PCA) that had been applied to all the spike waveforms from the corresponding electrode group acquired during the training period (here the Running task), (6) each spike was classified as coming from a given unit with respect to unit clusters that had been defined from spike sorting the training period data. Importantly, the PCA and classification model, used for identifying the unit origin of each spike, were determined offline by analyzing and curating the training data on a PC. The resulting PCA transformation matrix and classification model for each electrode group were manually inspected, then transferred to the FPGA for fast, online processing (*41*). In the FPGA, the PCA matrix converts online-detected spike waveforms into waveform features, which are then classified by comparing them to pre-determined reference features for each unit. The time elapsed from spike detection to the completion of spike classification is a deterministic latency of 1 ms for each spike, regardless of the number of spikes or units due to the real-time processing power of FPGAs (*40*, *41*).

Behavioral training

Acclimation to equipment

To acclimate the rats to wearing the harness and behaving on the treadmill, a series of steps were followed. First, the rats were subjected to water restriction for 1-2 weeks. Then, they began a habituation process in which they wore the jacket for 10-15 minutes each day, then the jacket plus belts, until they became comfortable with them. This typically took about 10 days. Then they were placed in the full harness on the treadmill for a few days with ample water provided from the lick port. During this time, they were encouraged to start obtaining water regularly from the lick port. This acclimation process helped the rats become accustomed to the harness and treadmill, which allowed for goal-directed running and BMI task behavior.

Training using the Running task

After acclimation, animals were exposed to a cue-rich VR environment (1 × 1 m square arena with 20 cm high walls) with proximal cues (on the floor and walls) and distal cues (around the arena above the walls). A tall, thin, spiral pillar (of maximum extent 20 × 20 × 80 cm) was used as the goal cue for all tasks. To increase its visual salience, the goal cue moved up and down at a frequency of approximately 1 Hz. During the initial training phase, the goal cue was placed close to the animal. Whenever the animal reached within 20 cm of the goal cue (center-to-center distance, called the "goal radius"), it was rewarded with 20-40 µL of water or 40-50 µL of sweetened water, and the goal cue was moved to a new location. This training phase served to help the rat understand that touching the goal cue leads to reward. Once the animals demonstrated consistent navigation to the goal cue, the Running task was introduced, in which the goal cue was placed at a random location at least 50 cm away from the location of the last reward, and the goal radius was decreased from 20 to 15 cm (or sometimes 10 cm). The same

VR environment was used for all of the training and experiments in order to allow the animal to form a well-learned map of the arena for the BMI experiments.

Surgery and electrode targeting

After an animal's performance in the Running task improved to the point of reaching the goal and obtaining reward ~200 or more times per 90 min training session, the animal was anesthetized with isoflurane and mounted in a stereotaxic frame for chronic implantation of electrodes. Two craniotomies were made, one over the CA1 field of the dorsal hippocampus of each hemisphere (centered at AP -3.7 mm, ML 2.8 mm). The dura was removed, and a 64-channel silicon probe consisting of 8 shanks with 8 recording sites each (Buzsaki64-H64LP_30mm, Neuronexus) was inserted into each hemisphere at an initial depth of ~900 μm. Each silicon probe was mounted on a shuttle drive (Nano Drive, Ronal Tool Company), which was fixed to the skull using OptiBond Universal (Kerr), Charisma A1 (Kulzer), dental cement (Jet Acrylic, Lang), and Calibra Universal (Dentsply Sirona). The probes were each connected to two of the 32-channel headstages (RHD2132, Intan Technologies), and the probes and headstages were surrounded by custom-made 3D-printed protective shells (the headstages remained with the implant when the animal was in its home cage). After a week of recovery, the electrodes were gradually adjusted over several weeks until they reached the CA1 pyramidal cell layer. Electrophysiological features such as the amplitude and polarity of sharp waves and the amplitude of spikes, recorded during each day's ~90 min training session in the Running task, were assessed visually to guide adjustment to the CA1 cell layer. After performing the BMI experiments, animals were anesthetized with isoflurane, small electrolytic lesions were made by passing anodal current (30 μA, ~10 s) through one electrode site per hemisphere, then animals received an overdose of ketamine and xylazine and were perfused transcardially with saline followed by 4% formaldehyde. Brains were coronally sectioned (50 μm thick) and placed on slides with mounting media containing DAPI (Vectashield) to verify recording locations.

Brain-machine interface tasks

Running task before either BMI task

After CA1 place unit activity was observed during a Running task training session (from offline sorting of data and analysis of spatially tuned firing, as in Fig. 1B), we started BMI experiments for that animal. On a given day, we first recorded neural activity for ~10 s to set the spike threshold (mean minus 4.5 to 5 × the standard deviation of the activity (*65*)). Then, animals were required to perform ~40 min of the Running task (~120 trials) while neural activity was recorded. Animals had to get to within 15 cm (or, in some sessions, 10 cm) of the center of the goal cue to get reward. Afterwards, animals were temporarily returned to their home cage, which was placed in the same room as the VR system.

Semi-automatic spike sorting

For the spikes that were detected online using threshold crossing per channel during the Running task, the four waveforms per spike from that electrode group (set of 4 adjacent electrodes) were saved, and these saved spike waveforms were used for offline semi-automatic

spike sorting (which was done separately per electrode group). The spike waveforms of an electrode group were PCA-transformed with 4 principal components kept, then a Dirichlet process Gaussian mixture model was applied (using the scikit-learn package in Python) to cluster the spike waveform features of each electrode group into 15-20 clusters (i.e., units). Using this "over-split" spike sorting model (i.e., one place cell might be split across more than one unit) reduces the amount of time needed to curate the unit clusters. This is important because a shorter curation time results in a smaller gap between the end of the training period (i.e., the Running task) and the start of the BMI task, thus reducing the potential impact of electrode drift. Additionally, using an over-split spike sorting model, which is related to clusterless decoding (*66*, *67*), leads to the same or better population decoding performance compared to single-unit spike sorting by maximizing the amount of information obtained from each electrode group. Finally, rapid manual curation was conducted using the interactive 3D visualization software of our BMI system, during which we separated noisy or unstable units from stable units by visual inspection, and a spike-to-unit classification model was built from this manually curated clustering result. We removed the noisy or unstable units from further processing (i.e., building the location decoder) while keeping these clusters for the online spike assignment to absorb noisy spikes. In this manner, many "noise" spikes were accurately labeled as belonging to noisy clusters and therefore did not contribute to either offline or online location decoding. However, some of the noise spikes (particularly when small electrode drifts occur) can invade the boundary of the well-isolated clusters. Such noise is likely inevitable, but we handle noise by explicitly training the location decoder to be less sensitive to noise in general (see below).

Spatial tuning (place fields), spatial information, and selection of place units

To determine the spatial tuning of a unit, a two-dimensional histogram of the unit's spiking locations in the 1 × 1 m arena (binned into 4 × 4 cm spatial bins) was first generated. The histogram was then normalized by the total duration the animal spent in each spatial bin. The resulting firing rate map was subsequently smoothed using a Gaussian filter with a standard deviation of 8 cm. Only periods of time when the rat's speed was >5 cm/s were used to calculate place fields. The collection of firing rate maps across the units represents a sample from the hippocampus' spatial map of the environment (cognitive map). Given the firing rate map f of a unit, the information rate I (bits/spike) of a unit was calculated as follows (*68*):

$$I = \int_x \frac{f(x)}{\lambda} \log_2 \frac{f(x)}{\lambda} p(x) dx$$

where λ is the mean firing rate of the unit and $p(x)$ is the probability that the rat is present in the spatial bin x .

Place units were selected from the pool of all stable units to train the decoder. Selection criteria included the peak rate of the unit's spatial firing rate map and the unit's information rate, both of which had to exceed certain thresholds, and which varied across animals (default parameters were set to exclude units with <0.5 Hz peak firing rate, spatial information <0.1 bits/spike, or <0.1 Hz or >4 Hz mean firing rate, the last of which should generally exclude interneurons, leaving units from pyramidal cells). The number of (oversplit) stable units and, of these, the number of stable place units satisfying the above criteria, were: for Jumper (157 and

68 units for rat 1, 373 and 122 for rat 2, 233 and 159 for rat 3, respectively), and for Jedi (253 and 110 units for rat 1, 288 and 124 for rat 2, 138 and 94 for rat 3). Note that only the place units were used for training the decoder, real-time decoding in BMI tasks, re-decoding analysis, and PV analysis. We used all stable units for the firing rate analysis in fig. S13 as well as for population burst event (PBE) detection.

Deep neural network decoder to estimate animal's current location in the Running task

To estimate the current location of an animal, we trained a 16-layer DNN (fig. S2) using the neural activity and locations during animal movement (counting only those periods when the speed, smoothed with a zero-lag 2 s boxcar window, was >5 cm/s) in the pre-BMI Running task. The DNN was trained to minimize the Euclidian distance between its estimate of the current location and the animal's actual current location (smoothed with a zero-lag 3 s boxcar window) every 100 ms. The input to the DNN consisted of the last 5 seconds of CA1 population activity, discretized into 50 100 ms time bins (note that in one session, the rat 3 Jumper session, the last 1.5 s of CA1 population activity was used instead as the input). Each bin contains the spike counts produced by N place units. To stabilize the spiking noise variance, the elements of the N × 50-dimensional (or N × 15-dimensional, when using the last 1.5 s of activity) spike count matrix were square-rooted, in accordance with methods proposed previously (*69*, *70*). The DNN transformed the N × 50-dimensional spike count matrix into a single 256-dimensional vector and passed it through all the internal layers until reaching the final linear layer, which output a 2-dimensional vector representing the x and y coordinates of the estimated animal location. Inspired by recent research that employs periodic nonlinear activation function (*71*), we designed a new network backbone that employs a sinusoidal activation function in place of the commonly used rectified linear unit (ReLU). We named this block Sine Net (fig. S2). We observed that a stack of this network structure using skip connections between each instance of the Sine Net significantly accelerated the training on some of our data in comparison to other nonlinear activation functions.

Data augmentation to improve decoder robustness

To improve the robustness of our DNN location estimation in the presence of input noise (which could come from online spike sorting, motion artifact, electrode drift, physiological state changes, and other sources), we employed data augmentation (*33*) that adds independent and identically distributed (IID) noise to the training data. This noise was generated using a Bernoulli random variable and a uniform random variable (between 0 and a maximum value). The Bernoulli variable introduced a probability of 0.5 that the spike count of each place unit at each time bin would be changed (either increased or decreased) independently. The uniform variable determined the magnitude of this change, which was sampled independently for each unit and each bin. To ensure that the input remained valid, we set the spike count to zero whenever it became negative after the change. During training, the DNN was exposed to various levels of noise by using the absolute value of a sinusoid as the maximum magnitude of the noise across epochs (fig. S2F). This data augmentation procedure allowed the network to learn to ignore independent noise across units (i.e., "off-manifold" activity). By training the DNN model to produce similar outputs in the presence of various amounts of noise, we improved its

robustness and generalization performance. (Note that data augmentation is only used during training but not the evaluation of decoder performance.)

For the Jedi task (rats 1-3) and one session of the Jumper task (rat 3), factor analysis (FA)-based reconstruction of the spike count matrix was conducted before adding the independent noise. FA is a dimensionality reduction technique widely used in BMI (*30*, *69*, *72*) that models shared variability and maps the population neural activity onto its intrinsic manifold. Akin to PCA, FA is used when the underlying structure of the data is believed to be a linear combination of uncorrelated latent variables, called factors, but, importantly, FA separates the neural population activity into two components: shared-variability that is generated by a set of low-dimensional factors (on-manifold activity) and independent noise that can be different across units (off-manifold activity). This explicit separation provides an additional source for data augmentation during training which could further improve our decoder robustness. To achieve this, latent factors (40-dimensional) were used to reconstruct raw spiking activity by removing off-manifold activity, ideally resulting in only on-manifold activity, then independent noise was added across units to create an additional set of training data. Because FA reconstruction (using the first 40 factors) reduces off-manifold activity in the raw input, this further encourages the DNN to rely more on the "on-manifold" activity (*30*) for location estimation. Our FA-based data augmentation was implemented in Python using scikit-learn and PyTorch.

Evaluation of decoder performance

We used 70% of the data from the Running task to train the current location decoder and tested the performance of the decoder on the remaining 30% of the data. Time intervals when the subject was stationary were excluded from both the training and test datasets. This procedure took ~10-20 min. After training, the decoder was evaluated on the test set using the coefficient of determination (i.e., R^2 score). To compute the R^2 score, the decoder output was first smoothed with a zero-lag 3 s boxcar window (as was the actual current location in the test set). (Note that the decoder output was not smoothed during DNN training.) The mean R^2 score was calculated by taking the mean of the R^2 scores for the x and y axes separately and was used as the final performance score of each individual session (Fig. 2C). A higher value of R^2 indicates more accurate decoding.

If the performance of the decoder on the test data had a mean R^2 score >~0.8, we proceeded with one of the BMI tasks: we uploaded the spike sorting model parameters to the FPGA, placed the animal back into the VR system, had the animal run 20 more trials (i.e., 20 different goal cue locations) of the Running task to visually verify the performance of the decoder, then started the BMI task (Jumper or Jedi). Note that on a given day, after the Running task, usually one session, but sometimes two sessions, of the Jumper or Jedi task were run (but the Jumper and Jedi tasks were generally not run on the same day). Rats 2 and 3 were first exposed to the Jumper task, while rat 1 was first exposed to the Jedi task (Table S1).

Deployment of DNN decoder to control VR in real time

Spike counts from each unit were recorded and binned in real time into 100 ms bins based on their FPGA timestamps. The square roots of these binned spike counts, organized into a matrix with dimensions N × 50 (or N × 15), were sent to the trained decoder every 100 ms for location estimation. The real-time spike binning and decoding were implemented in Python

and communicated to the VR game/task engine Playground using the PyTorch multiprocessing module, with each process running on a dedicated CPU and sharing data through shared memory. The parameters of the DNN were fixed after training, except for the running mean and variance in the batch normalization layer, which were updated every 60 s during the BMI tasks. The entire process of binning and decoding consistently took less than 50 ms on a PC (Dell Precision Tower 7910 with 40 CPU cores and 128 GB RAM), well within the 100 ms update interval. To smooth out any sudden jumps of the output to be rendered during BMI tasks, a 3- or 2-second moving average (i.e., an average of the last 30 or 20 outputs of the decoder) was applied to the output of the DNN to determine the updated location of the animal (in Jumper) or controlled object (in Jedi), respectively. This moving average introduced a delay of a few seconds in the BMI trajectory of animal or object relative to the current decoded locations, which can be seen in the supplementary video (movie S1). For both tasks, the new location that was determined every 100 ms and a command string were sent to our VR game engine to update both the task states and the VR rendering. The VR update typically took 1-2 rendering frames to complete. Note that the VR projector runs at 60 Hz and Playground (the VR engine) sends updates to the MouseoVeR VR system at 20 Hz, while the decoder updates the animal or object location at 10 Hz.

BMI navigation task ("Jumper")

In the Jumper task, which is a BMI version of the Running task, the animal's location in the virtual arena was decoupled from the treadmill movement; instead, animals were teleported to the average decoded location of the latest 3 s of decoder outputs (i.e., 30 consecutive decoding windows) every 100 ms. Whenever the rat reached within 15 cm (or, in some sessions, 20 cm) of the center of the goal cue, the current trial ended, the cue disappeared, a reward (20-40 µL of water or 40-50 µL of sweetened water) was delivered, and the goal cue reappeared at a new, random location at least 50 cm away from the animal's location, as in the Running task. The goal radius was fixed once the session started and remained the same for all trials in that session. If the animal failed to reach a given goal cue within 62 s, the trial was also considered ended, and the location of the goal cue was changed, at which point the next trial began.

BMI object location control task ("Jedi")

In the Jedi task, animals were virtually fixed at the center of the arena, the goal cue was placed at least 30 cm away from the center, and a visible (or invisible) controlled object (a rectangular object 20 × 20 cm wide and 30 cm tall with large, open sides and floating 5 cm above the floor) was teleported every 100 ms to the average decoded location of the latest 2 s of decoder outputs (i.e., 20 consecutive decoding windows). Whenever the controlled object remained within 15 (or, in some sessions, 20 cm) of the goal cue, reward could be triggered. Each reward pulse lasted 10 ms (0.3-0.4 µL of water or 0.2-0.3 µL of sweetened water) for each 100 ms decoding step, but a refractory period was implemented 0.3 s after reward delivery was triggered (i.e., after every 3 pulses). The duration of the refractory period followed a uniform distribution between 0 and 2 s. As in Jumper, the goal radius was fixed once the session started and remained the same for all trials in that session. For each trial, the location of the goal cue was fixed at a given location for ~3 min (187.4 s) or until the animal received at least 0.5 mL of

reward, whichever occurred first. After that, the trial ended, and the goal cue's location was changed while the controlled object’s location remained the same, thus starting the next trial, at which point the rat needed to control the external object to enter the new goal region.

Rationale for Jumper and Jedi task design

The Jumper task was designed to assess whether animals can use BMI to navigate to arbitrary goal locations in a goal-directed and model-based manner. To demonstrate this, animals should navigate toward each goal without searching other locations in the arena. Otherwise, animals would likely need to search the arena by producing random activity (random in the sense that it is not model-based, directed activity) until the decoded location reaches the current goal. Since the goal region only accounts for less than 7.1% or 12.6% of the arena for a 15 cm or 20 cm goal radius, respectively, not being able to use a world model (i.e., map of the environment) should result in BMI trajectories that search through many regions of the arena before finally reaching the goal and, thus, longer trial durations. Therefore, we believe performance in the Jumper BMI task is sufficient to determine whether animals can navigate using their learned world model in a goal-directed manner.

However, the Jumper task, which was designed as a first-person perspective game, makes it difficult to answer three additional and potentially important questions: first, whether animals can perform BMI navigation while remaining stationary; second, whether animals can hold remote locations in mind for extended periods of time, similar to what occurs in human mental time travel; third, whether animals can control an external object using the same world model (here, the same spatial map) as when controlling their own location during BMI navigation. The continuous updating of the arena view and associated optical flow in the Jumper task encourages animals to move. It also encourages animals to quickly activate representations of successive locations on the way to the goal. In addition, because the trial ends immediately when the goal is reached, this tends to limit opportunities to observe prolonged periods of activation of a given remote location.

Therefore, we designed the Jedi task as a third-person perspective game that differs from Jumper in two key ways. First, the view of the arena is stable since the animal’s location is fixed at the center of the arena. Such a fixed first-person perspective view will not produce forward-moving optical flow, and thus may reduce animal movement. Second, a Jedi trial does not end when the goal cue is reached, which thus allows for a much longer trial duration (here, up to 3 min) compared to Jumper trials (which typically last ~15 s) and allows for repeated and/or continuous activation of the representation of the goal location. These two features enabled us to observe whether animals can activate and maintain non-local representations around the goal region while remaining stationary for extended periods of time. Indeed, rats can be stationary for tens of seconds (i.e., as long as several Jumper trials) while performing the Jedi task, and they can also hold the decoded location (again, without physical movement) near or within the goal region for several seconds as well, as shown in the examples in Fig. 3B, Fig. 4H, fig. S12, and movie S1.

Post-experiment data analysis

Data were analyzed in Anaconda Python 3.8. All confidence intervals are 95% CIs. Two types of analyses are used in this study: "in-experiment analysis" and "post-experiment

analysis". In-experiment analysis was conducted after the Running task then applied to the BMI experiment, and is described above (e.g., semi-automatic spike sorting, selection of place units, training the DNN decoder, evaluating the decoder performance). Post-experiment analysis was conducted after the experiment and is described below.

The animal's BMI performance in the Jumper task

The performance in the Jumper task was assessed by the duration of trials compared to a randomized goal control (Fig. 2G). In an individual Jumper trial, a new goal cue is randomly generated away from the previous one when the last trial has finished (i.e., the animal reaches the previous goal and reward is delivered). The new goal cue may be placed behind the rat or within the rat's visual field. When the cue is generated behind the rat, the animal cannot at first see where the cue is and typically initiates the trial by turning its body to search for the cue after consuming the previous reward (during reward consumption the rat is stationary). The time between finishing the reward and turning the body to search for the goal cue is variable, as this behavior is self-initiated by the rat. For example, the rat can groom between trials. To accurately quantify the trial duration, the trial start is defined as the moment the animal starts to engage in the task during that trial. Specifically, when the cue is presented behind the animal, the trial start is defined as the moment the rat's body orientation changes by more than 12 degrees per second, as the rat needs to turn its body to search for the cue. When the cue is presented within the visual field of the rat and the rat does not turn to approach the goal cue, the trial start is defined as three seconds after the last trial ended, during which time the rat typically finishes consuming the reward. The trial duration is defined as the time elapsed from the trial start to the moment the goal cue is reached by the rat. To determine if the animal's behavior in Jumper was more goal-directed than chance would predict, we performed 1000 independent shuffled goal simulations and compared the mean trial duration of each simulation with the actual mean trial duration. In each simulation, the goal locations were randomly shuffled for each trial while the animal's BMI trajectories remained unchanged from the original data. A simulated trial ends when the BMI trajectory reaches the randomly shuffled goal cue (i.e., within the goal radius) and the simulation ends when the BMI trajectory of the entire session has been completely used. We then compared the mean trial duration of the actual Jumper session (containing ~50 trials) to the distribution of simulated mean trial durations. The p-value was estimated from the Z score of the actual mean duration compared to the shuffle distribution. A low p-value from this test (along with a shorter actual mean trial duration) indicates that the animal's behavior in the Jumper task was significantly more goal-directed than chance would predict.

As a further measure of goal-directed control, we computed the angle between the instantaneous direction of movement along the BMI trajectory and the direction from the current location to the center of the goal. The trajectory along each trial was divided into ~20 samples equally spaced in time during the trial. For each sample, the angle between the direction from the last movement step and the direction from the current location to the goal's center was computed. The distribution of angles from all samples of all trials were plotted in polar coordinates. An analogous distribution was computed for all Running trials. Both Jumper and Running task distributions were concentrated around a value that was near 0 degrees (where 0 represents movement directly toward the center of the goal). Interestingly, when the peak of the Running task angle distribution for an animal was shifted slightly away from 0, that animal's

Jumper task angle distribution was similarly shifted (Fig. 2H), which indicates that animals preferred to approach goals in a similar manner when running and during BMI behavior.

In addition, performance was assessed by the duration of trials compared to a shuffled unit control. In each of 200 independent shuffles, the spike trains of each unit were randomly assigned (once across all trials) to different units, the shuffled data was input into the original decoder, and the resulting BMI trajectory determined. Then the simulated durations of each trial were computed based on how this trajectory reached the original sequence of goals locations. We then compared the mean trial duration of the actual Jumper session to the distribution of simulated mean trial durations, and estimated a p-value based on the Z score. A low p-value (along with a shorter actual mean trial duration) indicates that the animal's performance in the Jumper task depended on the specific activity of place units, as opposed to non-spatially-specific modulation of the aggregate activity across units.

The animal's BMI performance in Jedi tasks

The performance in the Jedi task was evaluated using the Euclidean distance between the controlled object and the goal cue compared to a randomized goal control (Fig. 3C). The trial duration is not used as a performance metric in Jedi as a trial does not end when the object reaches the goal the first time, and a trial can last up to 3 min even if the controlled object is statistically close to (but not always within) the goal region. Like in Jumper tasks, periods of non-task-engagement, such as grooming or "trying to run out of the arena" (i.e., periods of constant running into a wall), are excluded from the analysis. Additionally, because the Jedi task was intended to evaluate the animal's ability to control a remote object while remaining as stationary as possible, low angular movement was utilized as an indicator of task engagement, and therefore periods during which the rat's body orientation changed by more than 12 degrees per second were excluded from the analysis. Note that, after removing these excluded periods, the majority of the time the animal did not move the treadmill (Fig. 3D). After excluding such non-engagement periods, a 2D histogram (with 2 × 2 cm spatial bins, smoothed by a 4 cm Gaussian kernel) of the decoded location distribution or controlled object location distribution (which were virtually identical, see fig. S11) during each trial was plotted as a visual assessment of how close the object was to the goal. To determine whether the decoded locations were closer to the goal locations than chance would predict, we compared decoded-location-to-goal distances to those from 1000 independent simulated control experiments. In each simulation, we first randomly selected a starting frame within the first 7 seconds of the Jedi session. Then, we subsampled the animal's decoded location every 7 s (i.e., every 70th frame). This 7 s interval ensured that the decoded locations were independent of each other because the decoding window was 5 s and we used an additional 2 s moving average for determining the location of the controlled object. For each subsampled decoded location, a random goal location was generated at least 30 cm from the center of the arena (this is because in the actual Jedi experiment each goal was at least 30 cm from the center). The distance between the decoded location and the goal location was determined and 15 cm was subtracted and, if the value was less than 0, the result was set to 0. This was done because it reflects the true distance from the decoded location to the goal as far as receiving reward is concerned by accounting for the goal radius. Then, for each simulated session (1000 simulations) and the actual Jedi session, a mean distance to goal averaged across all the of the subsampled decoded locations was calculated. The actual session's mean distance to goal was then compared to the distribution of

1000 simulated mean distance to goal values. The p-value was estimated from the Z score of the actual mean distance compared to the distribution of simulated mean distances. A low p-value (along with a smaller actual mean distance) indicates that the animal's behavior in the Jedi task was significantly more goal-directed than chance would predict.

In addition, performance was assessed by the object-goal distance compared to a shuffled unit control. In each of 200 independent shuffles, the spike trains of each unit were randomly assigned (once across all trials) to different units, the shuffled data was input into the original decoder, and the resulting BMI object locations determined. Then the simulated object-goal distances were computed between these object locations and the original sequence of goal locations. We then compared the mean distance for the actual Jedi session to the distribution of simulated mean distances and estimated a p-value based on the Z score.

Re-decoding of Jumper or Jedi experiment with shorter decoding window

To estimate the effect of a shorter decoding window on decoded locations, the experiments using a 5 s decoding window were re-decoded by using the original decoder, but using as input the most recent 1.5 s (15 100 ms bins) of spike count data at each time point and setting the first 3.5 s (35 bins) of data in each window to 0.

Re-decoding of Jumper or Jedi experiment with population bursts events (PBEs) excluded

To investigate the contribution of population burst events (PBEs) to the high performance of navigation in Jumper trials in which animals did not move the treadmill, or the high performance of controlling remote objects in the Jedi experiments, we detected and excluded these PBEs then applied the location decoder to the remaining neural activity and assessed the task performance post-hoc. A histogram of all place units was created with 10 ms bins and smoothed using a Gaussian kernel (with a 10 ms standard deviation). Segments where the peak of the smoothed histogram exceeded the mean plus 1.8 standard deviations were identified as candidate PBEs, with the start and end boundaries determined when the smoothed histogram crossed its mean value. Candidate PBEs of less than or equal to 10 ms duration were not counted as PBEs to reduce false positives, and the rest were counted as PBEs. Finally, the spike counts of all units in any 100 ms bin that overlapped with any PBE were set to zero, the original decoders used in each BMI experiment were then used to re-decode the data, and the navigational trajectory (fig. S10) or spatial distribution of resulting controlled object or decoded locations with respect to the goal location (figs. S11 and S12) in each trial was computed.

Population vector analysis

The fact that our deep neural network (DNN) performed well during the Jumper and Jedi tasks suggests that animals can voluntarily generate goal-directed, non-local spatial activity. In addition, it suggests that the DNN generalizes well from the training set (physical navigation activity during the Running task) to new unseen data (navigation and object location control activity during the BMI tasks). To explore what information our DNN might be relying on for this generalization, we considered the evidence for one reasonable hypothesis, which is that the animal is generating, and the DNN is using, spiking patterns similar to the population vectors of the spatial firing rate maps for the units at each location (Fig. 4A). We first binned the arena into

4 × 4 cm spatial bins, as we did when calculating place fields. For each spatial bin, we constructed a reference population vector (rPV), which is a vector of the averaged firing rates of all units at that spatial bin during the Running task. Next, for each moment (T = 500 ms windows each 100 ms) during the Jumper and Jedi tasks, we calculated the Pearson correlation coefficient between the current PV (i.e., the vector of average firing rates of the units during that 500 ms) and the rPV of the DNN's current decoded location. For comparison, we also calculated the Pearson correlation coefficient between the PV at each (T = 500 ms) moment in the Running task (using the 20 additional Running task trials after the animal was returned to the VR system and immediately before the BMI task started, which were not included in spike sorting or decoder training, or used to compute the rPVs) and the rPV of the animal's current location in the environment, which provides a benchmark for the "maximum" PV-rPV correlation values that can be expected when taking into account the natural variability of neural activity (especially over a timescale of 500 ms). Note that for the Running and BMI task PVs, we first performed a global normalization of firing rates so that the overall firing rate of each unit during the post-sorting/decoder-training/rPV-determination period (consisting of the 20 additional Running task trials and the BMI task) was scaled to match the overall rate of that unit during the Running task period that was used to determine the rPVs. We then compared the distribution of correlation coefficients across all moments during a session (Jumper, Jedi, or Running task) to the distribution of coefficients between the PV at each moment and the rPVs of random locations. Our results show that instantaneous PVs corresponding to each decoded location in Jumper or Jedi displayed significant (versus random levels of) similarity to the rPVs at those locations (Fig. 4B,C and fig. S14) and matched the highest similarity achieved by Running task PVs when considering a range of time windows (T = 0.5-5 s, Fig. 4D,E). This is consistent with the animal generating PVs for each location that are similar to the rPVs for those locations in the place field map, and with the DNN extracting that PV information to estimate the animal's current location in the Running task and generated location in the BMI tasks.

Assessing DNN decoder response to rPVs with and without noise

Although DNNs are essentially black boxes, to further test the hypothesis that our DNN decoder had been trained to detect activity patterns that are similar to the place map rPVs, we examined the DNN decoder's response to a single, constant, noiseless rPV input, where each rPV is associated with a known location. The trajectory in the Running task was used to provide a series of locations to test over. For each location in the trajectory (at each 100 ms step), there is an associated rPV. An identical copy of that current location's rPV was placed in each of the last 5 bins (500 ms) of the 50-bin (for 5 s decoding window) or 15-bin (for 1.5 s decoding window) input to the DNN, with the first 45 or 10 bins, respectively, set to 0. The R^2 score between the decoded outputs and actual locations were computed over the trajectory (with the results shown in Fig 4G with noise = 0). As a comparison, this same rPV was input into a Bayesian decoder (*45*) and the R^2 score determined. Both the DNN and Bayesian decoder accurately decoded noiseless rPV inputs, with high R^2 scores in each case. This demonstrates that the "ideal" PV input (the rPV for a location) is sufficient to have the DNN output that location, without requiring any temporal activity patterns. To assess and compare the noise robustness of both decoders, we tested their performance on rPVs with added noise. The maximum amplitude of noise added ranged from A = 0 to 10 Hz. For a given A, a value was randomly drawn from a uniform distribution between [-A, A]. Five noise vectors were

independently sampled (i.i.d. for each unit and each 100 ms time bin) and added to the 5 duplicate rPVs for each of the 5 bins, with any resulting negative firing rates in a bin set to 0. The noise axis in Fig. 4G displays A/2, since this is the upper bound of the averaged noise applied to the rPV (it is an upper bound since the final rate values are bounded by 0). The 5 noisy versions of the rPV were input to the DNN (with the first 45 bins set to 0 and last 5 bins set to the noisy rPV) or averaged in the case of the Bayesian decoder. In these simulations, the DNN decoder was much more robust to noise than the Bayesian decoder (Fig. 4G), as the DNN was trained to be robust to noise using data augmentation.

Local field potential (LFP) and wavelet analysis

In our real-time FPGA-based NSP system, only band-pass filtered raw data within the frequency range of 500-3000 Hz was recorded. To recover the full-band raw data, a Wiener deconvolution, which was validated on both ground-truth and simulated data, was applied to the saved filtered raw data. Subsequently, one electrode in the pyramidal layer where many spikes from place units were detected was selected for LFP analysis per session. The LFP signal was obtained by applying a low-pass Butterworth filter (cutoff frequency of 400 Hz, order of 5) to the reconstructed raw data from the selected channel (sampled at 25000 Hz). LFP signals were then downsampled from 25000 Hz to 1000 Hz. Wavelet spectrograms were then obtained using complex Morlet wavelets (σ = 5) across a range of frequencies from 0-60 Hz for specific time segments. The magnitude of the spectrogram was Z-scored. For computing the power spectral density (Fig. 4I), the LFP traces were first Z-scored so that the resulting PSDs could be pooled across animals.

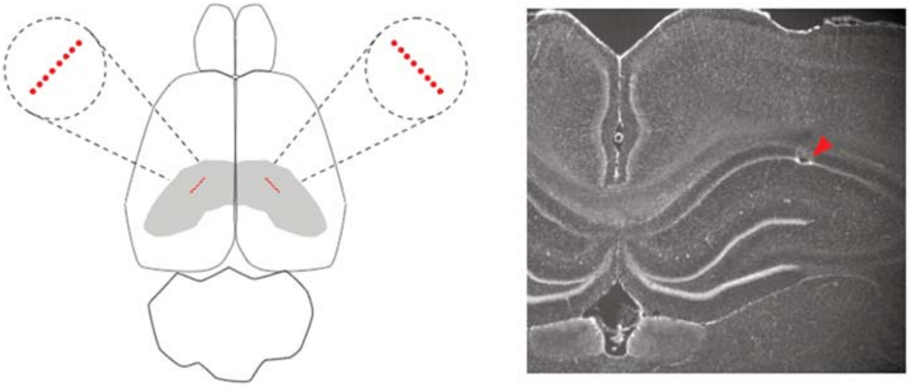

Fig. S1. Electrode locations. Schematic of location of silicon probe shanks (each red point represents a shank) with respect to top view of brain and hippocampus (shaded) (left). Example of electrode recording site location in hippocampal CA1 pyramidal cell layer (right).

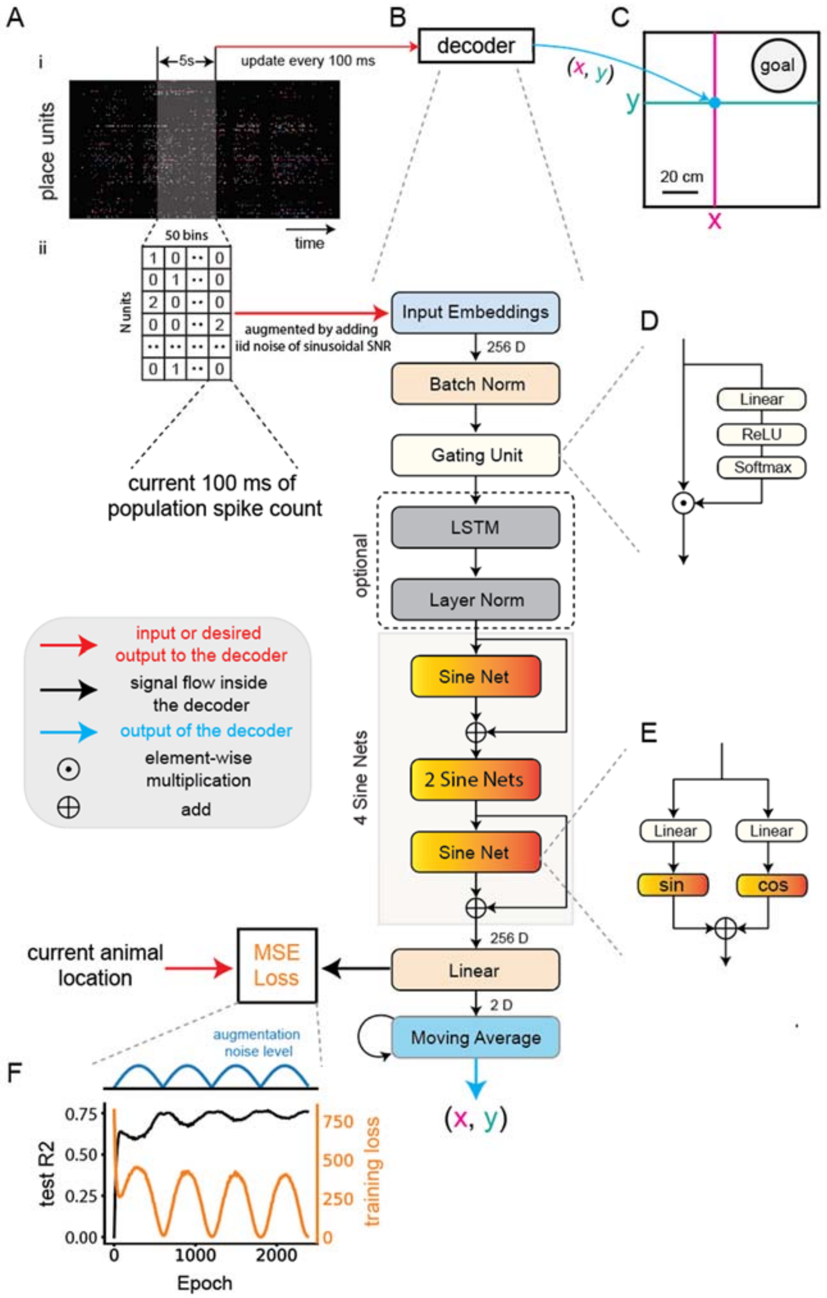

Fig. S2. Architecture and training of deep network that accurately decodes the animal's current location during the Running task and is used to translate hippocampal activity into control signals during the BMI tasks. (**A**) Population activity (spike counts per unit) in windows of 1.5 or 5 s were binned in 100 ms bins and input to the decoder. Windows were advanced in steps of 100 ms to give a series of inputs. (**B**) The layers and operations of the deep net decoder. (**C**) The outputs of the decoder were an x and y location value. (**D**) and (**E**) show details of gating unit and sine net layers. (**F**) The decoder was trained by minimizing the error between the current location of the animal (smoothed with a zero-lag 3 s boxcar window) and the decoder output. The training data was augmented by adding noise, which varied as shown as a sinusoidal function of training epoch. See methods for details.

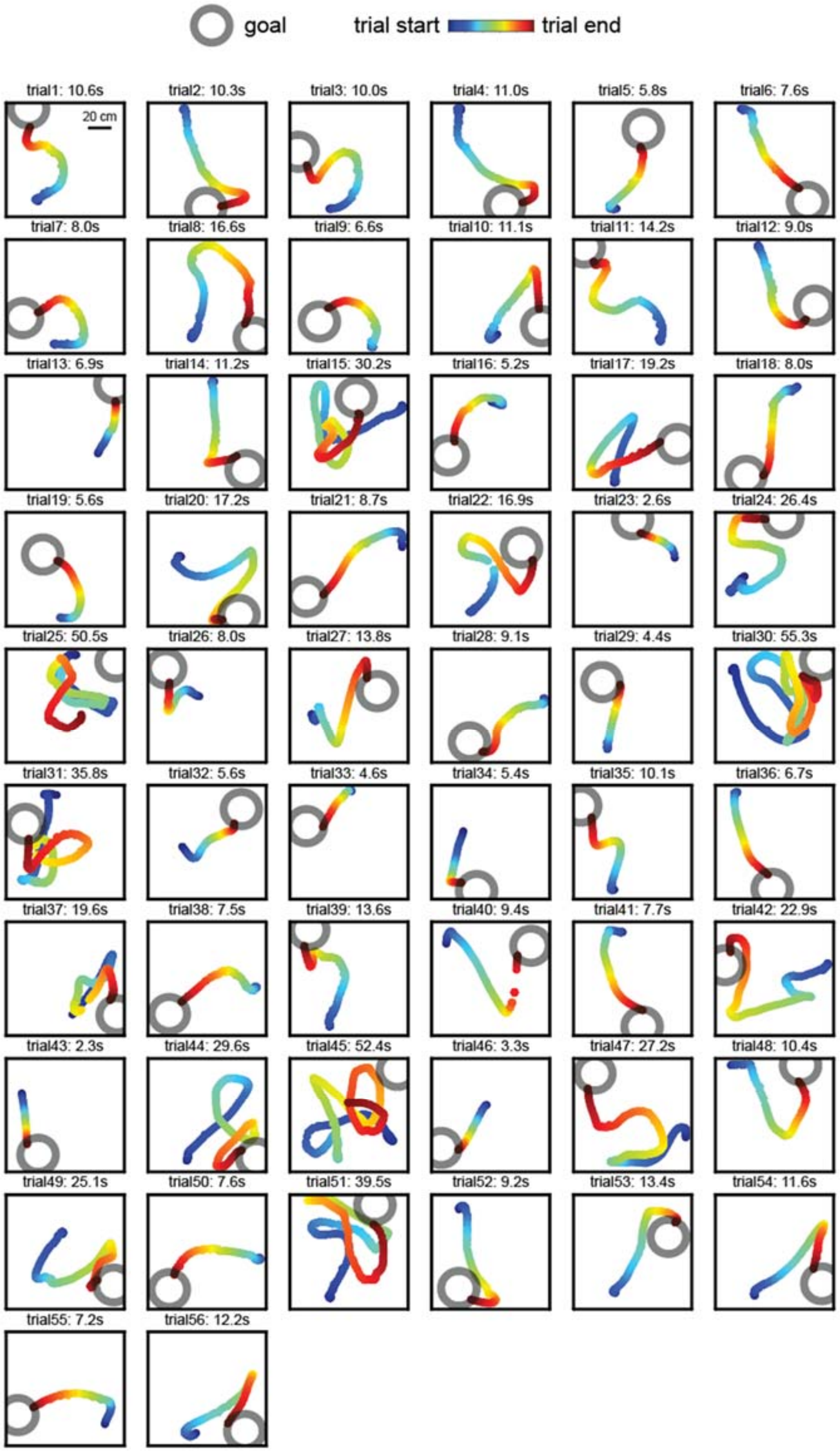

Fig. S3. All trials of the Jumper BMI experiment for rat 1. Note trials 1-12 and 53-55 are also shown in Fig. 2F.

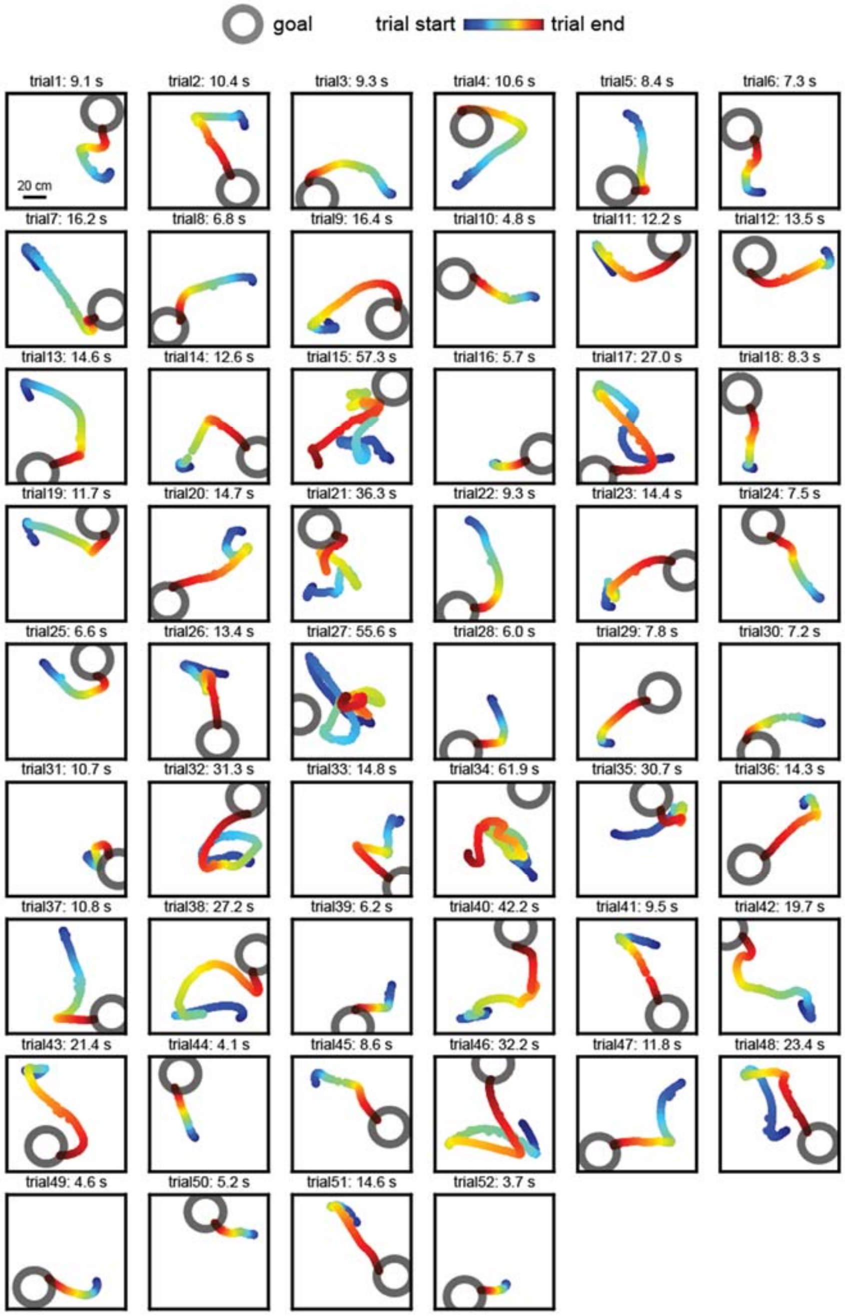

Fig. S4. All trials of the Jumper BMI experiment for rat 2.

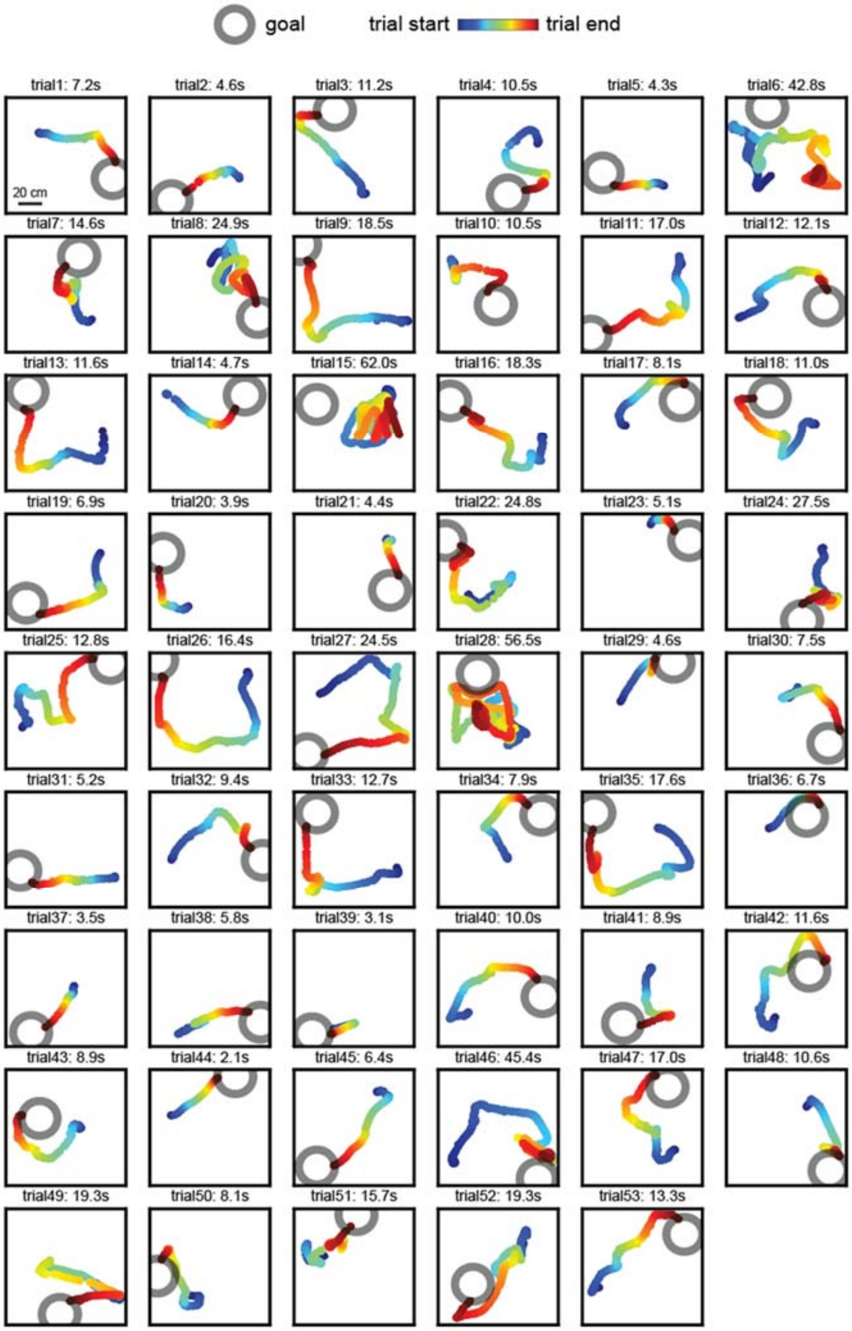

Fig. S5. All trials of the Jumper BMI experiment for rat 3.

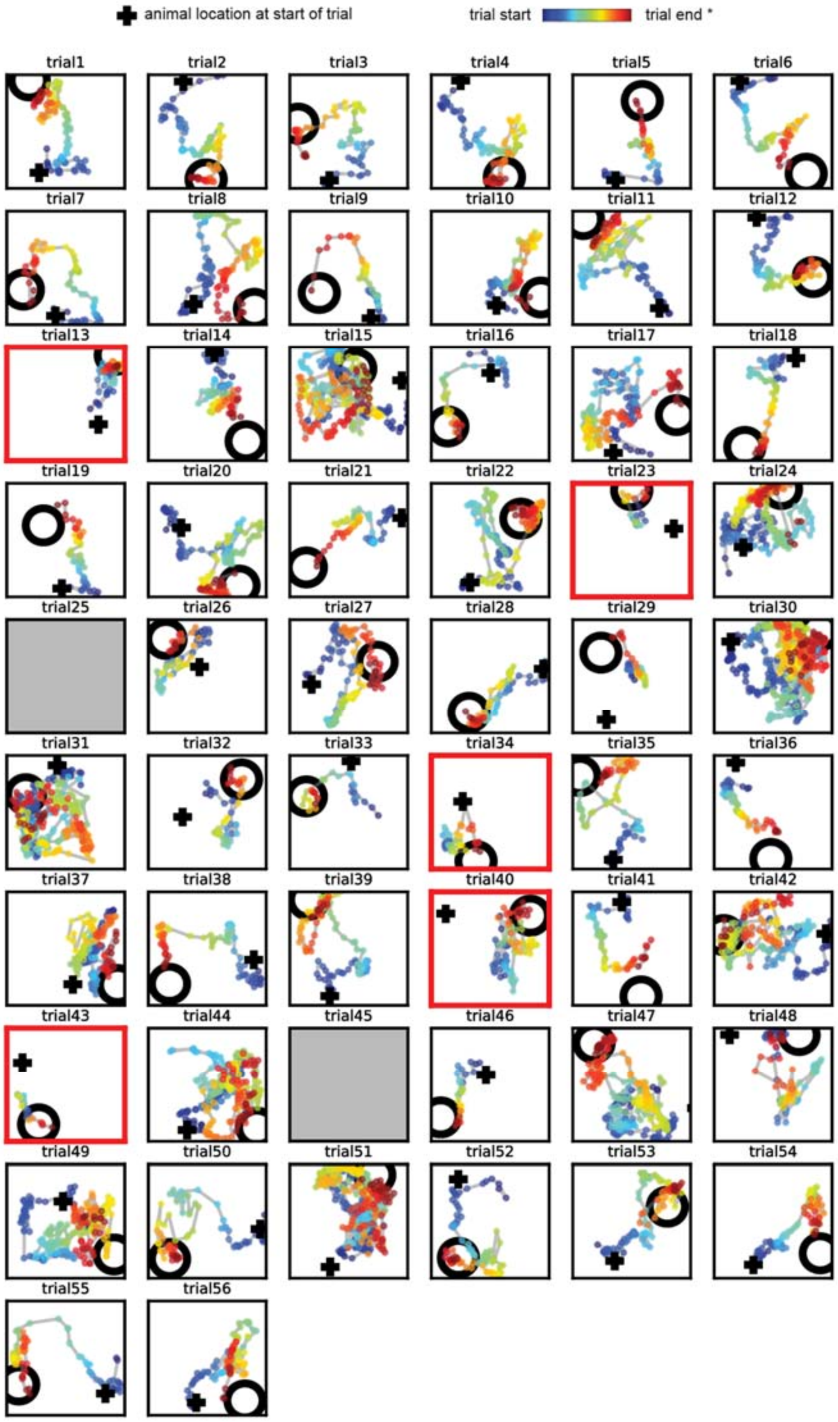

Fig. S6. All trials of the Jumper BMI experiment for rat 1 with locations re-decoded post-hoc using a shorter decoding window and without subsequent smoothing. Same trials as shown in fig. S3. Gray boxes: unsuccessful trials in which the animal did not reach the goal in time. Red outlined boxes: trials in which the animal did not move the treadmill. To re-decode the data, the original neural activity was run through the original decoder, except only the most recent 1.5 s of activity in each decoding window was used, while the first 3.5 s of activity was set to 0. The re-decoded location output every 100 ms was plotted as individual points here, without passing it

through the 3 s smoothing window used in the original experiment. The results show that the locations decoded based on more instantaneous activity yield similar trajectories to the original experiment (though with more jitter, as expected without smoothing). In a few trials—notably some of the non-treadmill-movement trials—at the start of the trial, these more instantaneously decoded locations were already near the goal and away from the animal. Note: In some cases, the decoded trajectories got near to but did not reach the goal. *In some cases, we removed the last few decoded locations in the trial because they passed through the goal at the end since the smoothing that was present in the actual experiment had been removed. Results were similar for rats 2 and 3 (fig. S7 and S8).

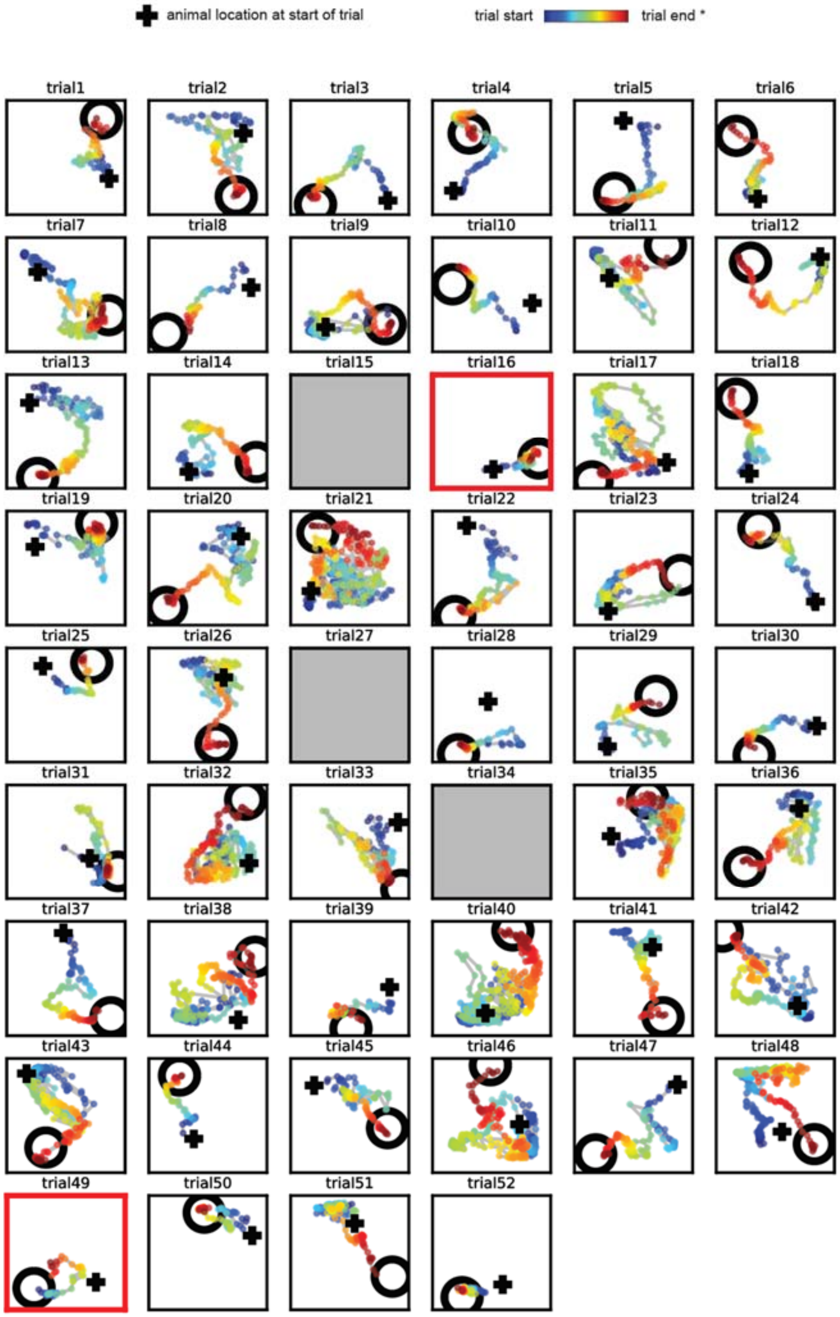

Fig. S7. Same as fig. S6 except for rat 2.

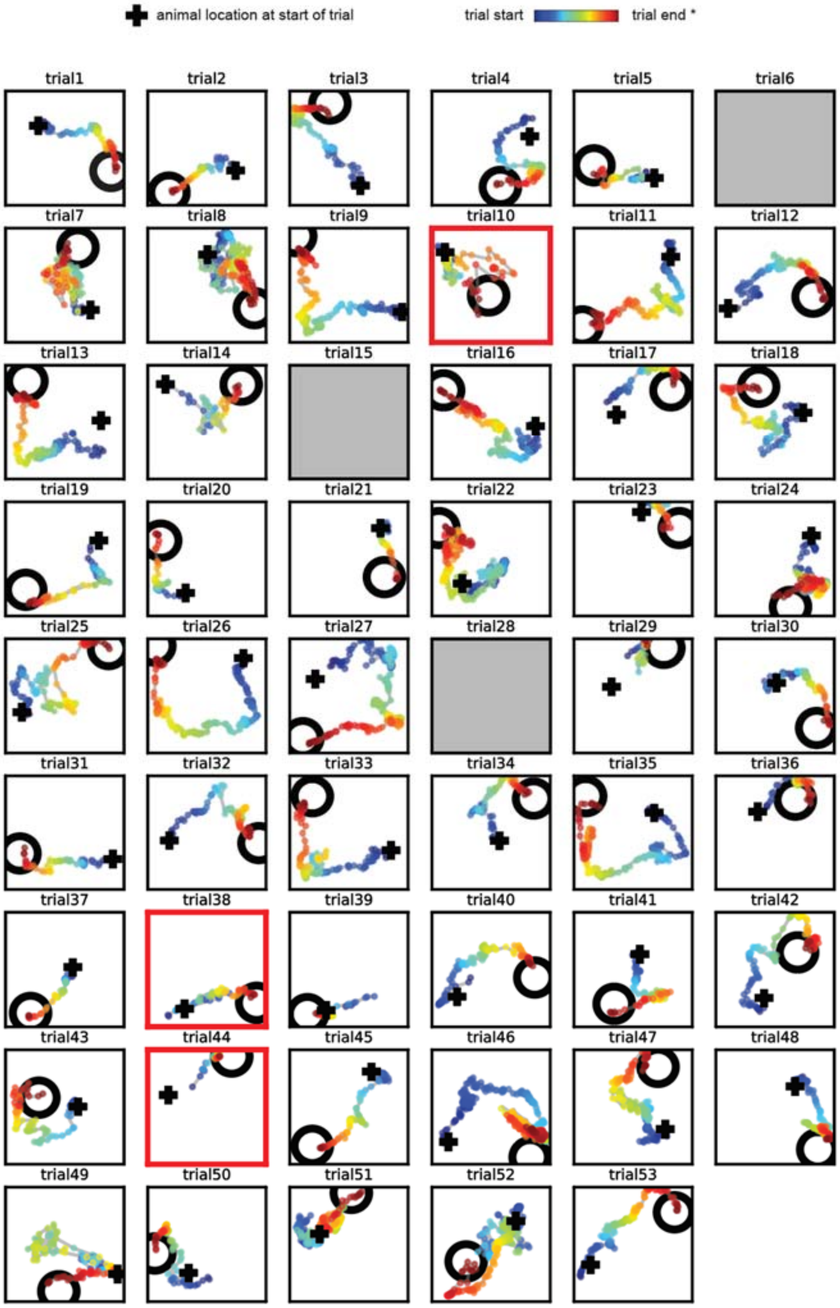

Fig. S8. All trials of the Jumper BMI experiment for rat 3 without smoothing of decoded locations. Note that, unlike for rats 1 and 2, the decoding window was 1.5 s in the original experiment for rat 3 so no re-decoding was necessary here. Otherwise, the description of what is displayed is the same as in fig. S6 and S7. Note that the trajectories jitter somewhat less than in fig. S6 and S7, likely because the decoding window here is the same window used in the original closed-loop experiment and the animal had learned to control its activity on this timescale.

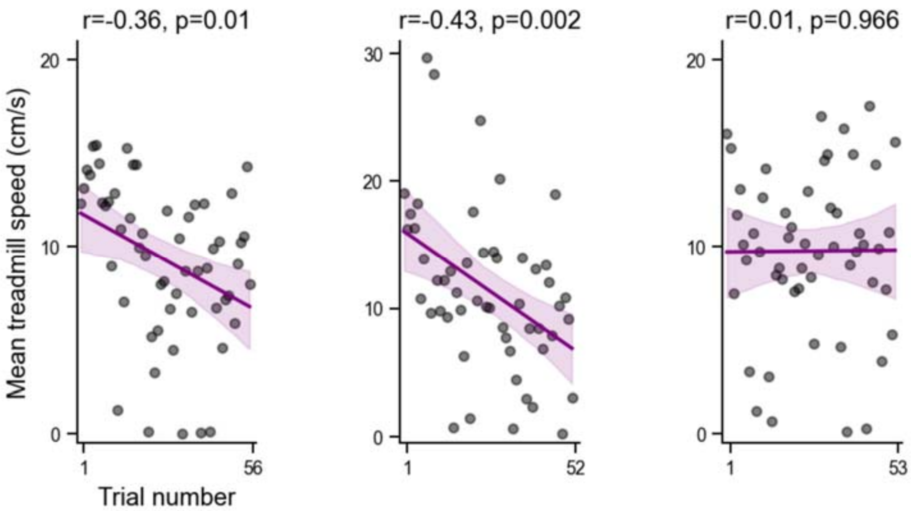

Fig. S9. Mean treadmill speed during each Jumper trial and linear regression for rats 1-3. Here and elsewhere all CIs are 95% CIs. For rats 1 and 2, treadmill movement decreased as the session progressed, while for rat 3 movement at the beginning was lower and remained at that level.

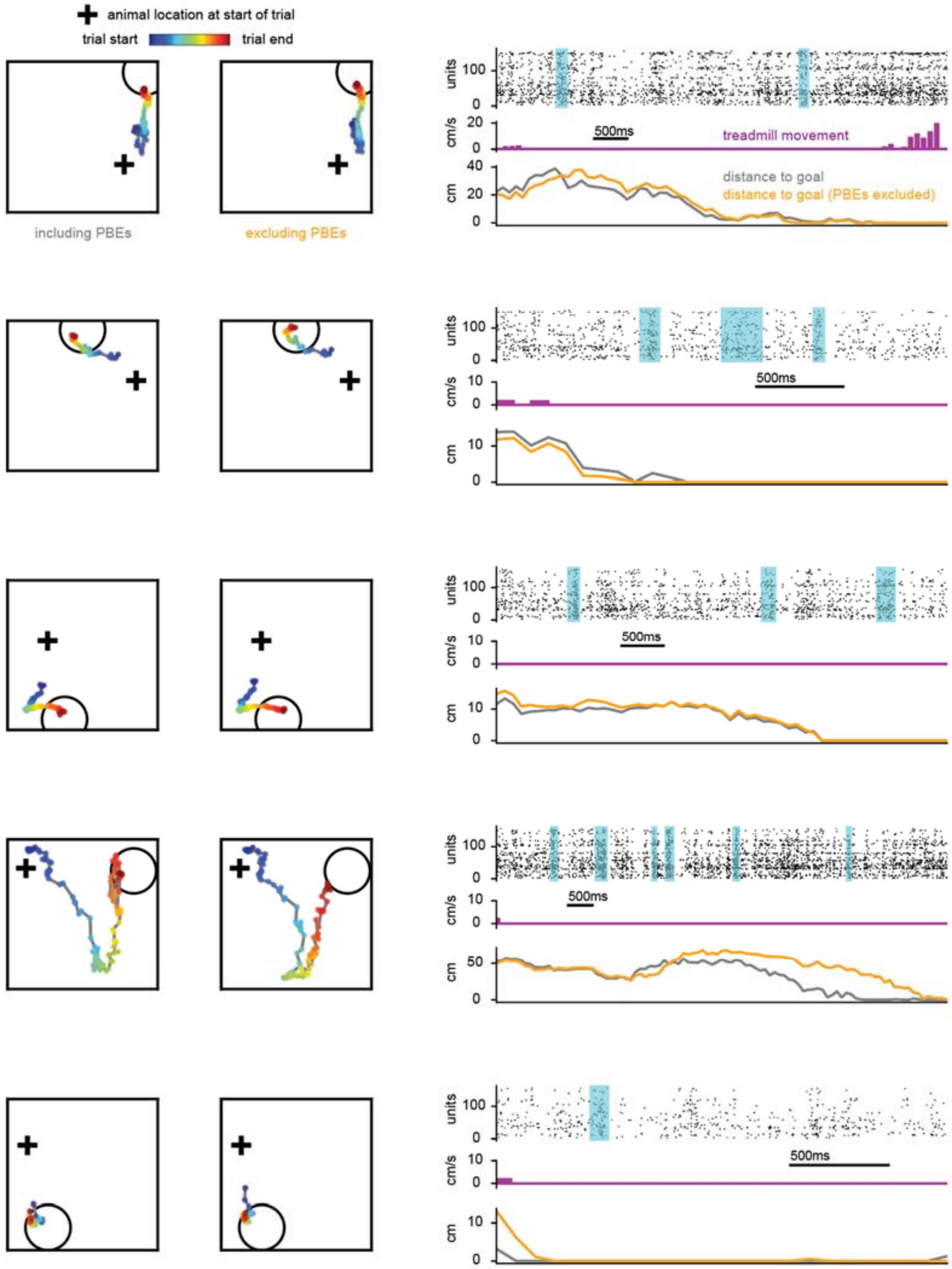

Fig. S10. All 10 Jumper trials during which there was little or no movement of the treadmill (continues on next page). In all of these trials the animal successfully navigated to the goal. Trajectory of decoded locations (column 1) and re-decoded locations after PBEs removed (column 2), showing each decoded location (every 100 ms, i.e. no smoothing). Right, from top for each trial: unit activity with PBEs (blue), treadmill speed, and distance of decoded location from goal. (Small differences in distance to goal in the 2 curves without visible PBEs are due to the effect of removing earlier PBEs on the running mean and variance which affect the input to the decoder.) The first 5 trials are from rat 1, the next 2 from rat 2, and the last 4 are from rat 3.

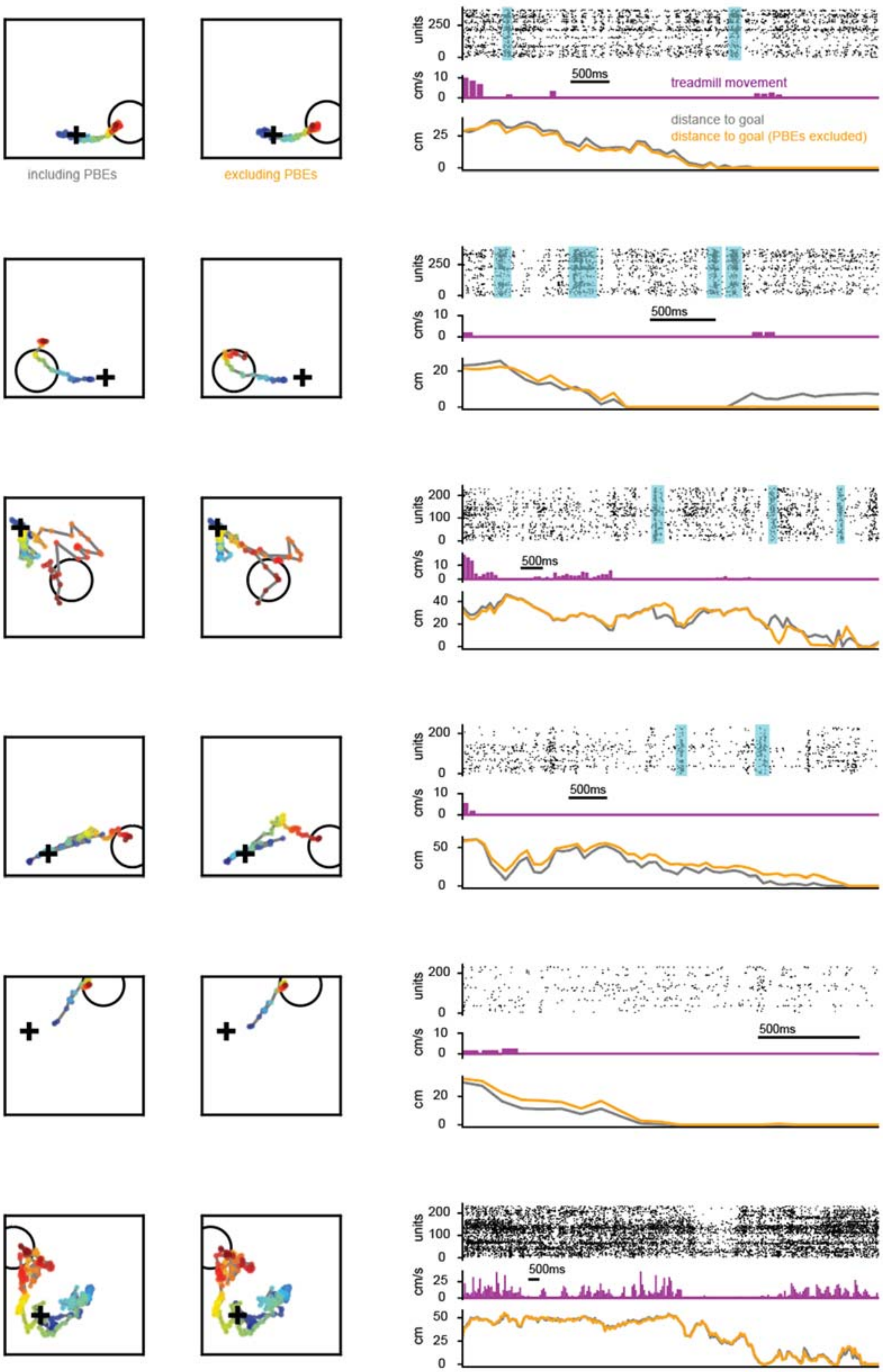

Fig. S10 (continued). The last (11th) trial contains significant treadmill movement, but the decoded location moves to the goal during a period of non-movement.

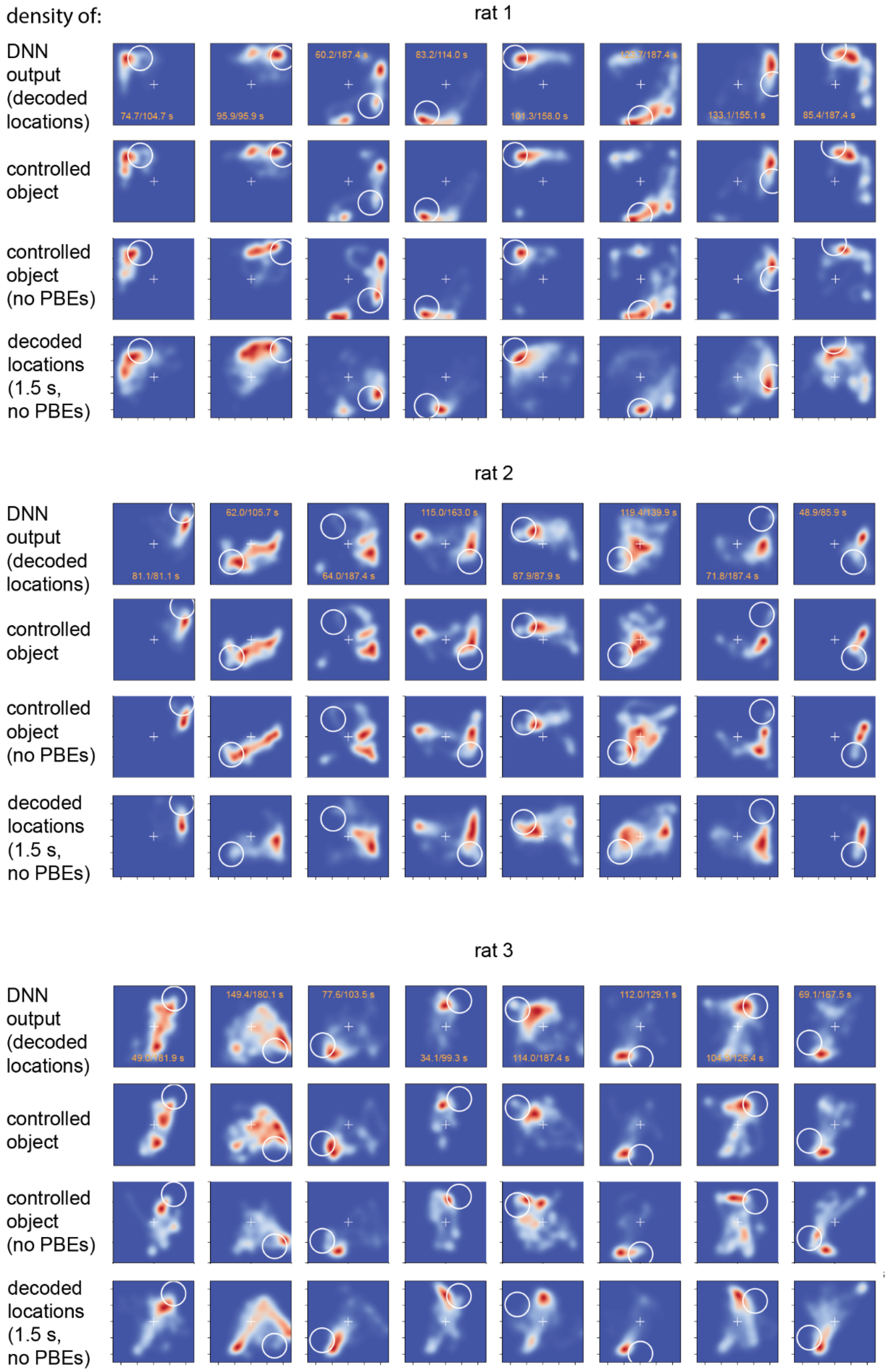
density of:
rat 1
DNN output (decoded locations)
controlled object
controlled object (no PBEs)
decoded locations (1.5 s, no PBEs)
74.7/104.7 s
95.9/95.9 s
60.2/187.4 s
83.2/114.0 s
101.3/158.0 s
133.1/155.1 s
85.4/187.4 s
rat 2
DNN output (decoded locations)
controlled object
controlled object (no PBEs)
decoded locations (1.5 s, no PBEs)
81.1/81.1 s
62.0/105.7 s
64.0/187.4 s
115.0/163.0 s
87.9/87.9 s
119.4/139.9 s
71.8/187.4 s
48.9/85.9 s
rat 3
DNN output (decoded locations)
controlled object
controlled object (no PBEs)
decoded locations (1.5 s, no PBEs)
149.4/180.1 s
77.6/103.5 s
34.1/99.3 s
114.0/187.4 s
112.0/129.1 s
69.1/167.5 s

Fig. S11. Distribution of real-time decoded location (i.e., DNN output), real-time controlled object location, controlled object location after excluding activity during population burst events (PBEs) during the Jedi task, and re-decoded location using only the most recent 1.5 s of activity in each decoding window (with the first 3.5 s of activity set to 0) and excluding activity during PBEs. For each animal, the four rows represent the same 8 consecutive trials. Top row per animal is the same as shown in Fig. 3A. Numbers in each panel indicate duration of trial excluding periods when animal's angular velocity was >12˚/s (a measure of task disengagement) (numerator) and total duration of trial (denominator), in seconds. The mean values for these were 88.3 s and 141.8 s (out of a maximum possible 187.4 s), respectively. In the bottom two rows per animal, spiking activity during detected PBEs was eliminated, then the location decoder was applied to the remaining data post-hoc. Comparison with the rows above shows that goal-directed control of the location of a remote object does not depend on this brief hippocampal population burst activity. Overall, the distributions are very similar across all conditions (rows).

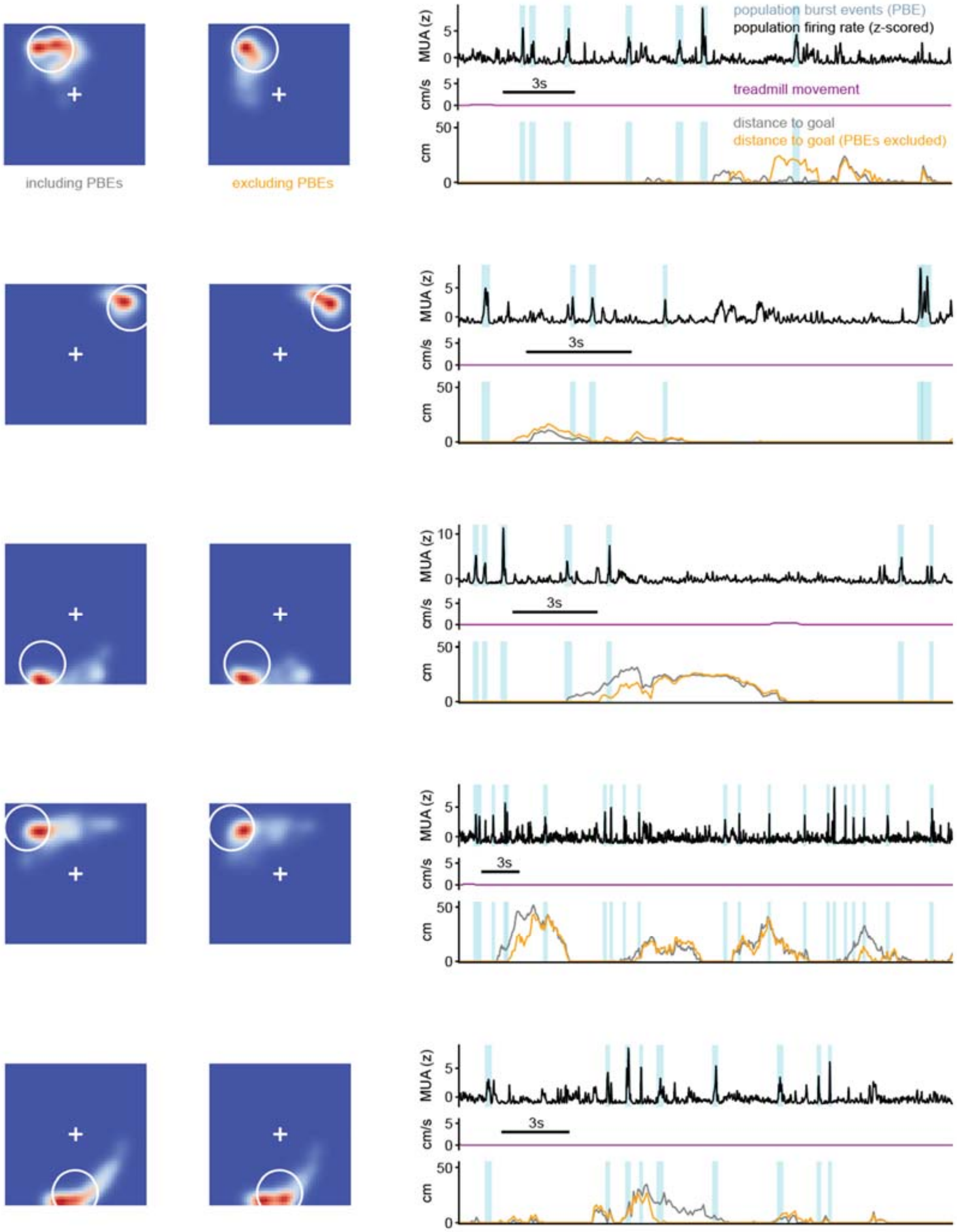

Fig. S12. Example longer segments during the Jedi task with little or no treadmill movement (continues on next page). There were 38 such segments in total (≥8 s long in which the 1 s-smoothed treadmill was ≤1 cm/s) and 11 are shown here (and Figs. 3B and 4H show 2 others). The animal successfully moved the decoded location to the goal and/or held it near there in 34 of the 38 cases. In 28 cases, PBEs were not required for this performance. In 4 cases performance was better if PBEs were included, and in 5 cases performance was better if PBEs were excluded. Distribution of decoded locations (column 1) and re-decoded locations after PBEs are removed (column 2). Right, from top for each trial: summed activity across all units with PBEs (blue), treadmill speed, and distance of decoded location from goal. Segments 1-6 are from rat 1, segments 7-8 from rat 2, and segments 9-11 from rat 3.

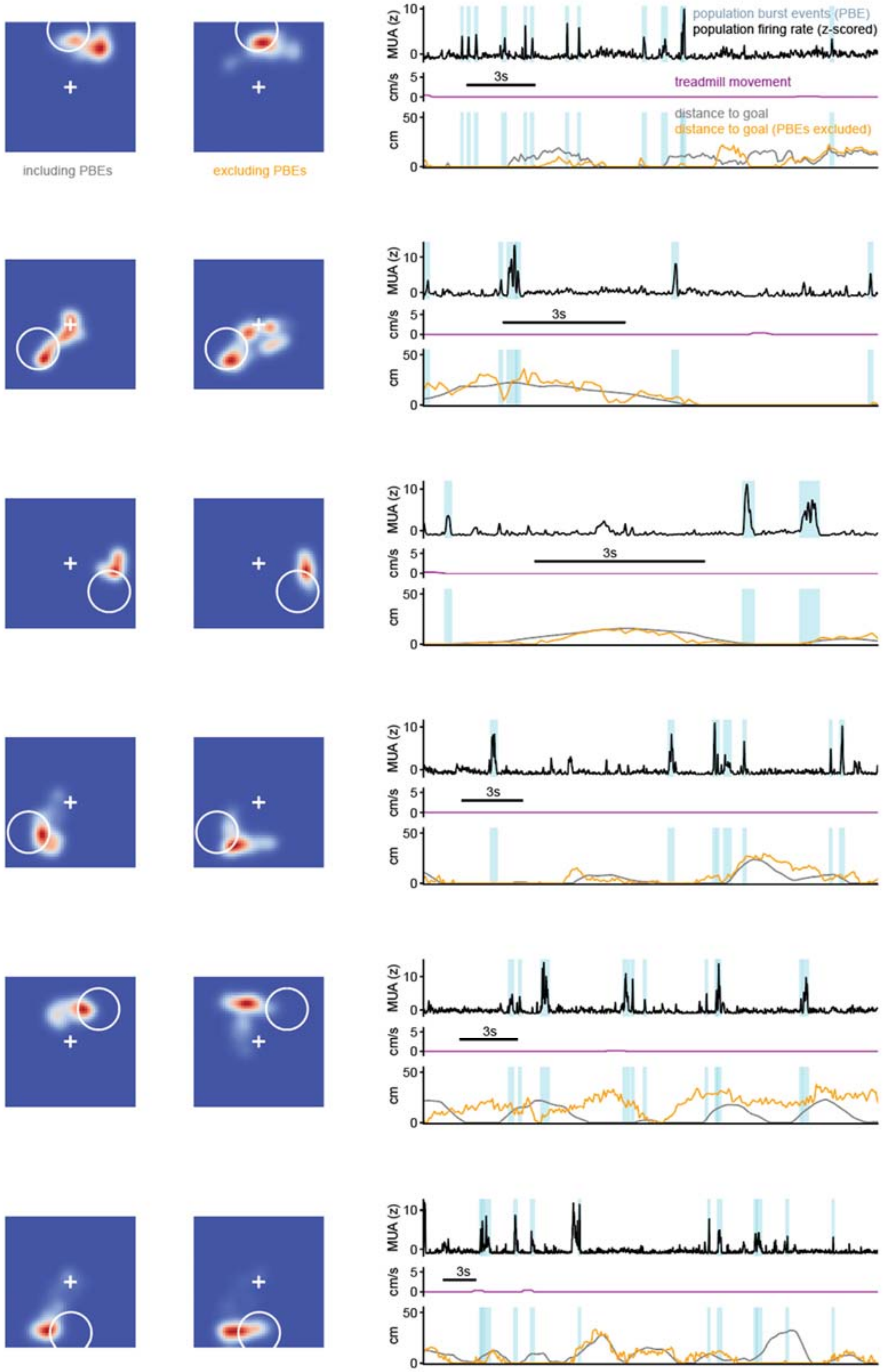

Fig. S12 (continued).

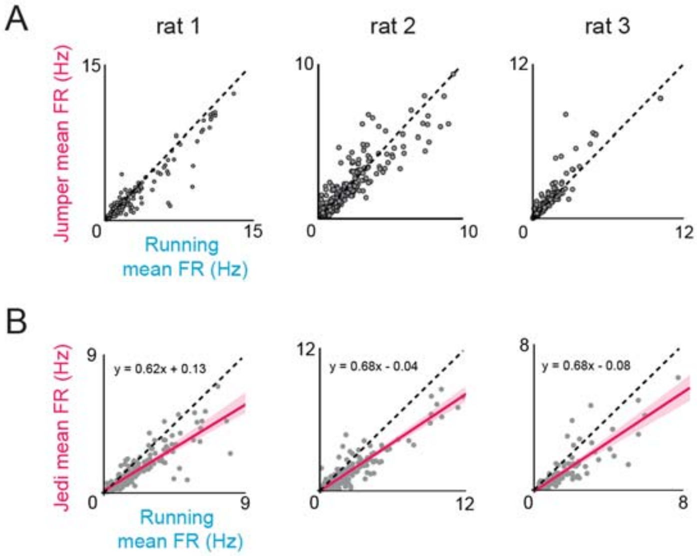

Fig. S13. (**A**) Mean firing rate of each unit during Jumper versus Running task trials. Dotted line: y = x. (**B**) Mean firing rate of each unit during Jedi versus Running task trials. Dotted line: y = x. Note that average firing rates are lower during the Jedi task compared to the Running task. For both (A) and (B), the data for the Running task came from the 20 Running task trials immediately before the BMI task (after spike sorting, decoder training, and the animal was returned to the VR system), which were the same trials as used for the PV analysis.

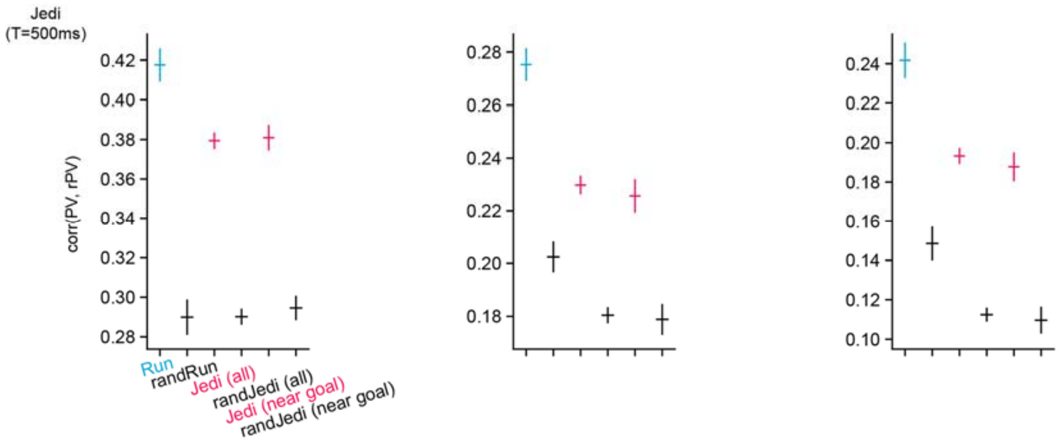

Fig. S14. Mean correlation of instantaneous (500 ms window) PV during Running or Jedi task with rPV for the current location (in Running task), current decoded location (in Jedi task), or random location in Running (randRun) or Jedi (randJedi) task for rats 1-3. In the "all" cases, the correlation was computed over all periods during the Jedi trial (except when the animal's body rotated >12˚/s). In the "near goal" cases, the correlation was computed only for those periods when the decoded location was also near (within 5 cm of) the goal. (The "near goal" correlations are the same as shown in Fig. 4C for "Jedi" and "randJedi".)

Rat	Day	BMI task	Goal radius (Running)	Goal radius (BMI task)	Decoding window	Reward	Goal cue
1	1	Jedi	10 cm			W & SW	visible
1	2	Jedi	10 cm	20 cm	5 s	W & SW	visible
1	3	Jedi		20 cm	3 s	W & SW	visible
1	4	Jumper	10 cm	20 cm	5 s	W	visible
1	5	Jumper					visible
1	6	Jedi	15 cm	15 cm	5 s	W	visible
1	7	Jedi	15 cm	15 cm	5 s	W	visible
1	8	Jedi	15 cm	15 cm	5 s	W	visible
**1**	**9**	**Jedi**	**15 cm**	**15 cm**	**5 s**	**W**	**visible**
1	10	Jedi	15 cm	15 cm	5 s	W	visible
**1**	**11**	**Jumper**	**15 cm**	**15 cm**	**5 s**	**SW**	**visible**
2	1	Jumper (2x)	10 cm	20 cm	5 s	W	visible
2	2	Jumper	10 cm	20 cm	5 s	W	visible
2	3	Jedi	10 cm	20 cm	5 s	W	visible
2	4	Jedi	10 cm	20 cm	5 s	W	visible
2	5	Jumper	10 cm	20 cm	5 s	W	visible
2	6	Jumper					visible
2	7	Jedi (2x)	10 cm	20 cm	5 s	W	visible
2	8	Jumper (2x)					visible
**2**	**9**	**Jumper (2x)**	**15 cm**	**15 cm**	**5 s**	**W**	**visible**
**2**	**10**	**Jedi (2x)**	**15 cm**	**15 cm**	**5 s**	**W**	**visible**
3	1	Jumper	15 cm	15 cm	5 s	W	visible
3	2	Jedi					invisible
**3**	**3**	**Jedi (2x)**	**15 cm**	**15 cm**	**5 s**	**W & SW**	**invisible**
**3**	**4**	**Jumper**	**10 cm**	**15 cm**	**1.5 s**	**SW**	**visible**

Table S1. BMI experiment details for rats 1-3 starting from the first day we ran these experiments up through the 3 Jumper and 3 Jedi experiments included in this study (bold). On some days the animal did 2 sessions (2x). For reward, W = water, SW = sweetened water. The goal cue was always visible for the Jumper task. Some parameters are missing from our records (blanks).

Movie S1. Illustration of VR system and environment, phases of experiment, and examples of Running task, validation of location decoder in Running task, Jumper BMI task, and Jedi BMI task, showing video of behavior, neural activity, and BMI output. Note that in Jumper and Jedi the location of the animal or controlled object, respectively, lagged the instantaneous decoded location. This was due to the 3 s (for Jumper) or 2 s (for Jedi) causal moving average used to smooth the BMI-generated trajectory and associated VR updates.
